# Diversity of root-knot nematodes of the genus *Meloidogyne* Göeldi, 1892 (Nematoda: Meloidogynidae) associated with olive plants and environmental cues regarding their distribution in southern Spain

**DOI:** 10.1371/journal.pone.0198236

**Published:** 2018-06-20

**Authors:** Antonio Archidona-Yuste, Carolina Cantalapiedra-Navarrete, Gracia Liébanas, Hava F. Rapoport, Pablo Castillo, Juan E. Palomares-Rius

**Affiliations:** 1 Instituto de Agricultura Sostenible (IAS), Consejo Superior de Investigaciones Científicas (CSIC), Avenida Menéndez Pidal s/n, Córdoba, Spain; 2 Departmento de Biología Animal, Biología Vegetal y Ecología, Universidad de Jaén, Campus ‘Las Lagunillas’ s/n, Jaén, Spain; Institute for Sustainable Plant Protection, C.N.R., ITALY

## Abstract

Root-knot nematodes of the genus *Meloidogyne* are recognised worldwide as a major production constraint in crops of primary economic importance. Knowledge of their diversity and prevalence, as well as the major environmental and agronomical cues for understanding their distribution in specific areas is of vital importance for designing control measures to reduce significant damage. We provide the first detailed information on the diversity, distribution and levels of *Meloidogyne* species infecting wild and cultivated olive soils in a wide-region in southern Spain that included 499 sampling sites. Overall *Meloidogyne* spp. were found in 6.6% of sampled olive plants, with 6.6% and 6.5% for cultivated and wild olive, respectively. We identified five previously described *Meloidogyne* spp. (*Meloidogyne arenaria*, *M*. *baetica*, *M*. *hapla*, *M*. *incognita*, *M*. *javanica*) and one new species (*Meloidogyne oleae* sp. nov.) which, characterized using integrative taxonomy, increases the known biodiversity of *Meloidogyne* spp. affecting olive. *Meloidogyne arenaria* and *M*. *incognita* were only found infecting cultivated olive varieties, while, *M*. *baetica* was only found infecting wild olive. Three major parameters drive the distribution of *Meloidogyne* spp. in cultivated olives in southern Spain, cover vegetation on alley, irrigation and soil texture, but different species respond differently to them. In particular the presence of *M*. *incognita* is highly correlated with sandy loamy soils, the presence of *M*. *javanica* with irrigated soils and cover vegetation, while the presence of *M*. *arenaria* is correlated with the absence of cover vegetation on alley and absence of irrigation. These parameters likely influence the selection of each particular *Meloidogyne* species from a major dispersal source, such as the rooted plantlets used to establish the orchards.

## Introduction

Root-knot nematodes (RKN) of the genus *Meloidogyne* Göeldi, 1892 [1] are recognised worldwide as one of the major production constraints of crops of primary economic importance, including vegetables, fruit-crops, ornamental and wild plants [2]. *Meloidogyne* species are among nature’s most successful plant parasites and have a significant economic impact on host-plants due to their wide host range and distribution throughout temperate and tropical environments [2]. However species determination of *Meloidogyne* spp. is complex, difficult and time-consuming even for experts. Identification of RKN species is essential for the design of effective nematode management strategies such as crop rotation and plant resistance. Recently 50 *Meloidogyne* species (about 50% of total species) have been characterized molecularly by ribosomal (D2-D3 expansion segments of 28S rRNA and ITS1 rRNA and partial 18S) and mitochondrial genes (*coxI*, *cox*II-16S), constituting a useful tool for molecular-based species identification [3–6]. Additionally, the analysis of isozyme electrophoretic patterns, in particular esterase (Est) and malate dehydrogenase (Mdh), have proven to be a valuable tool for precise identification of *Meloidogyne* species, particularly the most important tropical species (*M*. *incognita* (Kofoid & White, 1919) Chitwood, 1949, *M*. *javanica* (Treub, 1885) Chitwood, 1949 and *M*. *arenaria* (Neal, 1889) Chitwood, 1949 among others) [7]. In fact the application of molecular methods to studies of RKN and systematics has revealed that some long-assumed single species are cryptic species that are morphologically indistinguishable but may be phylogenetically distant to one another [8]. For these reasons, the integration of molecular and isozyme electrophoretic pattern techniques with classical morphological approaches should help to provide tools for differentiating *Meloidogyne* species and significantly improve and facilitate the routine identification of these nematodes.

*Meloidogyne* spp. and root-lesion nematodes (*Pratylenchus* spp.) are the most damaging plant-parasitic nematodes of cultivated olive (*Olea europaea* L. subsp. *europaea* var. *europaea*), especially in nurseries [9, 10]. Olive, in wild and cultivated forms, is widely distributed in the Mediterranean Basin, and particularly in southern Spain [11–14]. Wild (*Olea europaea* L. subsp. *europaea* var. *sylvestris*) and cultivated olives are hosts of and frequently suffer damage by RKN found in their rhizosphere, including *M*. *arenaria*, *M*. *baetica* Castillo, Vovlas, Subbotin, & Troccoli, 2003, *M*. *javanica*, *M*. *incognita*, *M*. *hapla* Chitwood, 1949, *M*. *lusitanica* Abrantes & Santos, 1991, *M*. *spartelensis* Ali *et al*. 2015 [15, 16]. Initially, with the aim of deciphering the biodiversity of *Meloidogyne* spp. infecting wild and cultivated olives in southern Spain, we surveyed 499 sampling points in Andalusia covering the diversity of olive cropping systems from traditional groves to new intensive orchards, as well as agroforestry stands where both olive forms were present. Our survey found 33 infested sampling points containing 35 populations of *Meloidogyne* species, morphologically similar to known *Meloidogyne* spp. The definitive identification of those populations prompted us to carry out an integrative taxonomic study to identify the species within this complex and economically important genus.

The suppression of the negative effects produced in crops by RKNs is considered as a difficult task due to the biological traits of both crop and RKN species as well as the high complexity of the soil environment [2, 17]. Understanding the drivers of community structure, however, is an essential point for increasing our knowledge regarding ecological phenomena involved in RKN management. In this context, recent studies have been performed on cultivated olives with the aim of finding the important factors influencing RKN population dynamics [18, 19]. Although both these studies suggested that the presence of RKN species could be determined by human activities (i.e. widespread introductions from nurseries), they also found habitat factors driving the distribution of RKNs among cultivated Moroccan olives [18, 19]. Nonetheless a general pattern for statistically significant environmental factors affecting RKN distribution in olive plants is lacking for Spain, which furthermore differs somewhat from African environmental conditions.

The general objective of this research was to study the occurrence and abundance of *Meloidogyne* species in olive plants in Southern Spain. More specifically our research aims were: *i*) to survey and then identify the *Meloidogyne* spp. populations detected in wild and cultivated olives; *ii*) to carry out a molecular characterisation of these *Meloidogyne* populations based on sequences of the D2-D3 expansion segments of the 28S nuclear ribosomal RNA gene, the ITS1 of rRNA, partial 18S rRNA, partial *cox*II*-*16S sequences and *cox*I; *iii)* to describe a new species *Meloidogyne oleae* n. sp. parasitizing wild and cultivated olives which was discovered during the species identification; *iv*) to study the phylogenetic relationships of *Meloidogyne* spp.; and *v)* to determine environmental patterns structuring the species composition of RKNs infecting cultivated olives in southern Spain.

## Material and methods

### Ethics statement

No specific permits were required for the indicated fieldwork studies. Permission for sampling the olive orchards was granted by the landowners. The samples from wild olives were obtained in public areas, forests, and other natural areas and do not involve any species endangered or protected in Spain, nor are the sites protected in any way.

### Study area, soil-sampling design and nematode extraction

**Fig 1:**
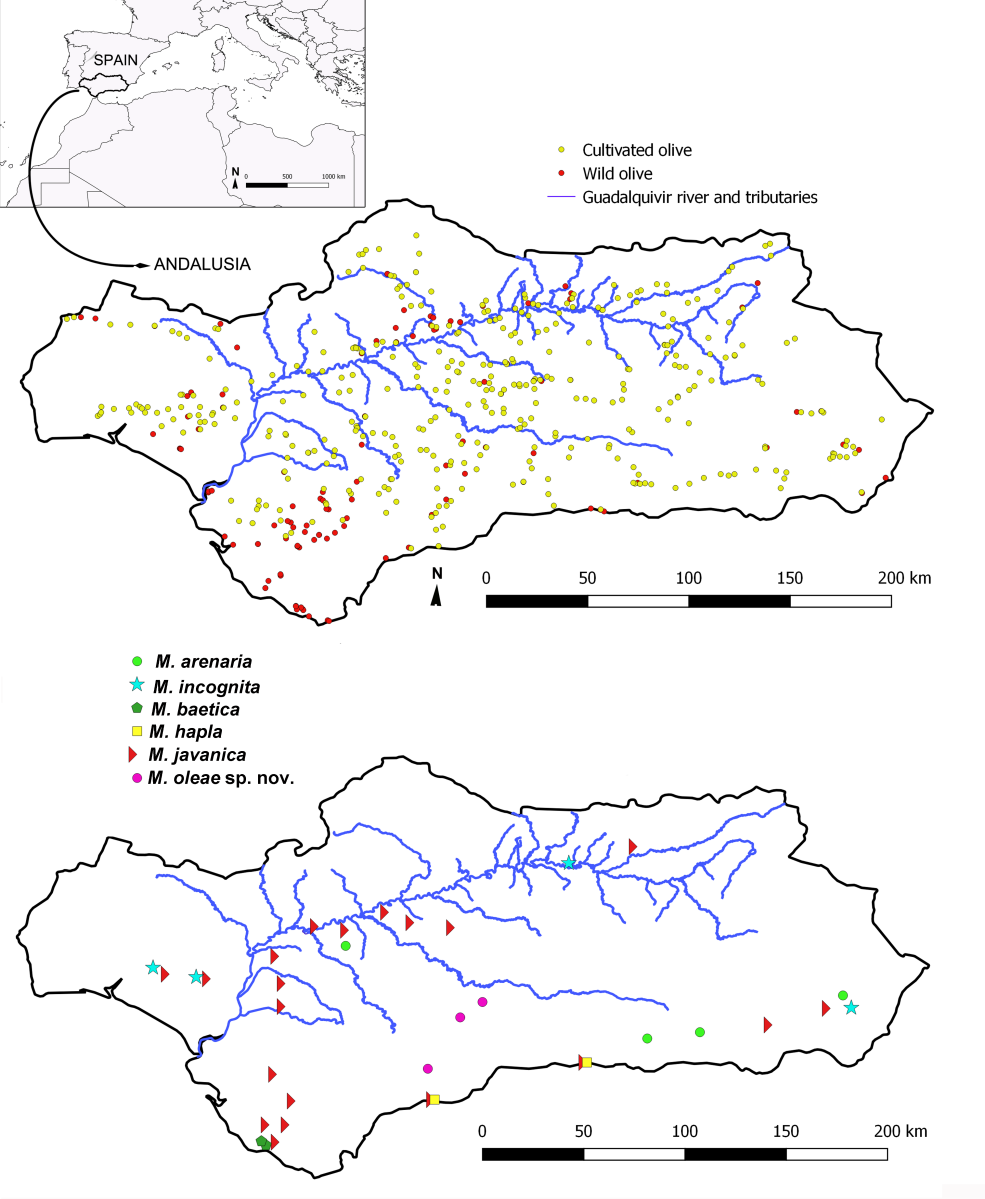
Geographic locations of all sample sites (upper map) and sample sites where root-knot nematodes of the genus *Meloidogyne* were found (lower map). This map may be similar but not identical to other published maps of Andalusia and is used only for the purpose of showing the sampling sites.

Nematodes were surveyed from 2012 to 2017 during the spring season in wild and cultivated olives growing in Andalusia, southern Spain (Table 1, Fig 1). A total of 123 and 376 sampling sites of wild and cultivated olives, respectively, were arbitrarily chosen in the eight provinces of Andalusia. We covered the diversity of olive growing systems, cropping systems including agroforestry stands, cropping systems from traditional groves to new intensive orchards, and agroforestry stands where both olive forms were present. The number of sampling sites was proportional to the area of wild and cultivated olive in each province (Table 1, Fig 1). Soil samples were collected and analyzed as described by Archidona-Yuste *et al*. [11, 20]. As described in detail previously [11], soil samples were collected with a hoe for nematode analysis from four to five trees randomly selected in each sampling site. The roots of the selected plants were carefully surveyed to a soil depth of 5 to 40 cm. The infected roots along with surrounding soil were put into polythene bags, properly labeled and brought to the laboratory of Nematology, IAS-CSIC.

**Table 1:** Taxa sampled for *Meloidogyne* species and sequences used in this study.

Species	Sampling site code	Administrative locality	host-plant	Density^●^	D2-D3	ITS1	18S	coxII-16S	coxI
**1. *M*. *oleae* sp. nov.**	AR107	Tolox (Málaga)	wild olive	121	MH011963	MH011973	MH011979	MG996751	MG996758
***M*. *oleae* sp. nov.**	AR107	Tolox (Málaga)	wild olive	121	MH011964	-	-	MG996752	-
***M*. *oleae* sp. nov.**	AR107	Tolox (Málaga)	rosebush	134	MH011965	-	-	-	-
***M*. *oleae* sp. nov.**	AR107	Tolox (Málaga)	lesser periwinkle periwinkle	18	MH011966	-	-	-	-
***M*. *oleae* sp. nov.**	AR107	Tolox (Málaga)	carob tree	71	MH011967	-	-	-	MG996759
***M*. *oleae* sp. nov.**	JAO28	Antequera (Málaga)	cultivated olive	4	MH011968	MH011974	MH011980	MG996753	-
***M*. *oleae* sp. nov.**	JAO28	Antequera (Málaga)	cultivated olive	4	MH011969	MH011975	-	MG996754	-
***M*. *oleae* sp. nov.**	JAO31	Antequera (Málaga)	cultivated olive	46	MH011970	MH011976	MH011981	MG996755	-
**2.** *Meloidogyne arenaria*	JAO65	Lanjarón (Granada)	cultivated olive	138	#	-	-	-	-
*Meloidogyne arenaria*	JAO77	Uleila del Campo (Almería)	cultivated olive	39	#	-	-	-	-
*Meloidogyne arenaria*	JAO78	Cádiar (Granada)	cultivated olive	2	#	-	-	-	-
*Meloidogyne arenaria*	JAO93	Fuentes de Andalucía (Sevilla)	cultivated olive	2	#	-	-	-	-
**3.** *Meloidogyne baetica*	AR025	Vejer de la Frontera (Cádiz)	wild olive	8	MH011971	MH011977	MH011982	MG996756	MG996760
*Meloidogyne baetica*	AR026	Vejer de la Frontera (Cádiz)	wild olive	14	*	*	*	*	-
**4.** *Meloidogyne hapla*	AR137	Maro (Málaga)	wild olive	9	*	*	*	*	-
*Meloidogyne hapla*	JAO41	Marbella (Málaga)	cultivated olive	2	MH011972	MH011978	MH011983	MG996757	-
**5.** *Meloidogyne incognita*	JAO76	Sorbas (Almería)	cultivated olive	124	#	#	#	#	-
*Meloidogyne incognita*	ST005	Niebla (Huelva)	cultivated olive	4	#	#	#	#	-
*Meloidogyne incognita*	ST007	Hinojos (Huelva)	cultivated olive	4	#	#	#	#	-
*Meloidogyne incognita*	ST020	Andújar (Jaén)	cultivated olive	5727	#	#	#	#	-
**6.** *Meloidogyne javanica*	AR001	Medina Sidonia (Cádiz)	wild olive	31	#	#	#	#	-
*Meloidogyne javanica*	AR002	Medina Sidonia (Cádiz)	wild olive	13	#	#	#	#	-
*Meloidogyne javanica*	AR003	Medina Sidonia (Cádiz)	wild olive	3	#	#	#	#	-
*Meloidogyne javanica*	AR005	Alcalá de los Gazules (Cádiz)	wild olive	1	#	#	#	#	-
*Meloidogyne javanica*	AR137	Maro (Málaga)	wild olive	49	#	#	#	#	-
*Meloidogyne javanica*	JAO41	Marbella (Málaga)	cultivated olive	8	#	#	#	#	-
*Meloidogyne javanica*	JAO70	Instinción (Almería)	cultivated olive	330	#	#	#	#	-
*Meloidogyne javanica*	JAO104	Alcolea del Río (Sevilla)	cultivated olive	34	#	#	#	#	-
*Meloidogyne javanica*	JAO106	La Campana (Sevilla)	cultivated olive	138	#	#	#	#	-
*Meloidogyne javanica*	JAO107	Utrera (Sevilla)	cultivated olive	34	#	#	#	#	-
*Meloidogyne javanica*	JAO112	Arcos de la Frontera (Cádiz)	cultivated olive	1	#	#	#	#	-
*Meloidogyne javanica*	JAO138	La Rambla (Córdoba)	cultivated olive	12	#	#	#	#	-
*Meloidogyne javanica*	ST043	Tabernas (Almería)	cultivated olive	80	#	#	#	#	-
*Meloidogyne javanica*	ST064	Écija (Sevilla)	cultivated olive	148	#	#	#	#	-
*Meloidogyne javanica*	ST076	Bollullos Condado (Huelva)	cultivated olive	3	#	#	#	#	-
*Meloidogyne javanica*	ST078	S. José Rinconada (Sevilla)	cultivated olive	51	#	#	#	#	-
*Meloidogyne javanica*	ST081	Aznalcázar (Sevilla)	cultivated olive	10137	#	#	#	#	-
*Meloidogyne javanica*	ST093	Utrera (Sevilla)	cultivated olive	88	#	#	#	#	-
*Meloidogyne javanica*	ST111	Fuente Palmera (Córdoba)	cultivated olive	8	#	#	#	#	-
*Meloidogyne javanica*	ST129	Vilches (Jaén)	cultivated olive	59	#	#	#	#	-

(-) Not obtained or not performed.

(*) Sequenced population but not deposited in GenBank database, since was identical to other sequences of the same species.

(#) Identified using the digestion of the partial *cox*II-16S [31]

● = Population density was calculated as the mean of *Meloidogyne* nematodes per 500 cm^3^ of soil.

Root-knot nematodes were extracted from a 500-cm^3^ sub-sample of rhizosphere soil and root samples by the centrifugal-flotation method [21]. In some cases in which a new taxa was detected there was insufficient material in the olive samples for suitable description. Thus, additional soil and root samples were collected from both olive and additional potential hosts located nearby, e.g. rosebush (*Rosa* sp.), lesser periwinkle (*Vinca minor* L.), and carob tree (*Ceratonia siliqua* L.). The soil adhering to the root samples was gently removed, and the roots were observed for root-knot nematode infection (presence or absence of galls). When roots of a sample were galled, the roots were stained with Phloxine B (15 mg/liter of tap water) for 20 min., and females and egg masses were dissected directly and used for identification and to assess the *Meloidogyne* spp. population density [22].

### Morphological studies

As described in detail previously [10], for diagnosis and identification, females were collected directly from wild and cultivate galled olive roots, while males, eggs and second-stage juveniles (J2) of nematodes were extracted from the rhizosphere by centrifugal-flotation [21] and from feeder roots by blending in a 0.5% NaOCl solution for 4 min [23]. Specimens for light microscopy (LM) were killed with gentle heat, fixed in a 4% solution of formaldehyde + propionic acid and processed to glycerin by Seinhorst’s rapid method [24]. Perineal patterns of mature females were prepared according to standard procedures [25]. Briefly, root tissues were teased apart with forceps and half spear to remove adult females. The lip and neck regions of the nematode were excised, and the posterior end was cleared in a solution of 45% lactic acid to remove remaining body tissues. Then, the perineal pattern was trimmed and transferred to a drop of glycerin. At least 20 perineal patterns were examined for species identification. Specimens were examined using a Zeiss III compound microscope with Nomarski differential interference contrast at powers up to 1,000x magnification. Randomly selected specimens of each life-stage were measured. Measurements and drawings were made at the camera lucida on glycerine infiltrated specimens. All measurements were expressed in micrometers (μm). All other abbreviations used are as defined in Siddiqi [26].

For scanning electron microscopy (SEM) fixed specimens were dehydrated in a gradient ethanol series, critical-point dried, sputter-coated with gold according to Abolafia *et al*. [27] and observed with a Zeiss Merlin Scanning Electron Microscope.

### Isozyme phenotype analysis

As described in detail previously [16], to obtain sufficient individuals of each *Meloidogyne* species population for electrophoretic analyses, the root-knot nematode populations under study and a reference *M*. *javanica* population from olive trees sampled at Córdoba, Spain [28], were increased on tomato (cv. Roma) in a glasshouse at 25 ± 3°C. For that, a single egg mass of each *Meloidogyne* species population was placed beneath the roots of individual tomato seedling in 12-cm pots filled with sterile loamy soil. However, since no reproduction occurred in tomato plants for *M*. *oleae* sp. nov. populations, five young, egg-laying females of *M*. *oleae* sp. nov. from galled roots of wild and cultivate olives were used for isozyme phenotype analysis [esterase (Est) and malate dehydrogenase (Mdh)]. For that, females were macerated in microtubes containing 5 μl of 20% (wt/vol) sucrose, 1% (vol/vol) Triton X-100 and 0·01% (wt/vol) bromophenol blue. Electrophoresis was carried out in 7×8-cm separating (pH 8·4) and stacking (pH 6·8) homogeneous gels, 7% and 4% polyacrylamide, respectively, 0·75-mm thick, with Tris-glycine buffer in a Mini Protean II electrophoresis unit (BioRad). Gels were stained with the substrate α-naphthyl acetate for Est and with Fast Blue RR (Sigma-Aldrich) for Mdh. Band patterns and relative migration of the bands (Rm) were compared to *M*. *javanica* [29].

### Histopathology

Galled roots from wild and cultivated olives infected by *M*. *oleae* sp. nov. populations, as well as from the additional hosts (rosebush and lesser periwinkle), were selected for histopathological studies. As described in detail previously [15], the roots were gently washed free of adhering soil and debris, and individual galls were selected as well as root segments of uninfected plants. Galled and healthy root tissues were fixed in FAE (formalin:acetic acid:60% ethanol = 2:1:17 vol/vol) for a minimum of 48 h, dehydrated in a tertiary butyl alcohol series (70, 85, 90, 100%), and embedded in paraffin. Embedded tissues were sectioned transversely at 10 to 12 μm with a rotary microtome, mounted on glass slides, stained with 0.05% toluidine blue O prior to paraffin removal [30], mounted permanently, and observed with an optical microscope. Images were captured with a Leica QW5001 image processing system.

### DNA extraction, PCR and sequencing

For molecular analyses, DNA of one female nematode of each RKN population was extracted and PCR assays were conducted as described by Castillo *et al*. [15]. The region of the mitochondrial genome between the cytochrome oxidase subunit II (*coxII*) and 16S rRNA mitochondrial DNA (mtDNA) genes was amplified for the identification of common root-knot nematodes (*M*. *arenaria*, *M*. *incognita*, *M*. *javanica*) and potential unknown RKN species, using primers C2F3 (5’-GGTCAATGTTCAGAAATTTGTGG-3’) [31] and MRH106 (5’-AATTTCTAAAGACTTTTCTTAGT-3’) [32]. The reference *M*. *arenaria*, *M*. *incognita* and *M*. *javanica* populations from olive trees [28] previously identified and maintained on tomatoes in the greenhouse served as a positive control throughout this study. Detailed protocols of PCR amplification for rDNA fragments and the mtDNA fragment were as described by Powers and Harris [31]. HinfI-digestion of amplified products was conducted in a 10 μl volume containing 5 μl of PCR product, 1 μl of HinfI restriction buffer (10X) and 15 units of HinfI (TaKaRa Biotech). Digestion was allowed to proceed for 3 h at 37°C. Restricted PCR products were separated on a 2% agarose gel. This pattern was compared as in Powers & Harris [31]. For samples not identified as the common root-knot nematode species, full PCR products were amplified and sequenced. Three ribosomal DNA (rDNA) fragments (D2D3 region of 28S rRNA, ITS, and 18S rRNA) and two mtDNA region (*coxI*, and coxII-16S) were amplified and sequenced. The D2-D3 expansion segments of 28S rDNA was amplified using the D2A (5’-ACAAGTACCGTGAGGGAAAGTTG-3’) and D3B (5’-TCGGAAGGAACCAGCTACTA-3’) primers [33]. The ITS region was amplified using forward primers 18S (5’-TTGATTACGTCCCTGCCCTTT-3’) and Vrain2R (5’-TTTCACTCGCCGTTACTAAGGGAATC-3’) [34].The partial 18S rRNA was amplified using primers 988F (5’-CTCAAAGATTAAGCCATGC-3’), 1912R (5′-TTTACGGTCAGAACTAGGG-3’), 1813F (5’-CTGCGTGAGAGGTGAAAT-3′) and 2646R (5′-GCTACCTTGTTACGACTTTT-3′) [35]. The *cox*II-16S was amplified using the primers mentioned above and finally, the portion of the *cox*I gene was amplified as described by Derycke *et al*. [36] using the primers JB3 (5´-TTTTTTGGGCATCCTGAGGTTTAT-3´) and JB5 (5´-AGCACCTAAACTTAAAACATAATGAAAATG -3´).

PCR products were purified and sequenced as described by Ali *et al*. [16]. The newly obtained sequences were submitted to the GenBank database under accession numbers indicated on the phylogenetic trees and Table 1.

### Phylogenetic analysis

D2-D3 expansion segments of 28S rRNA, ITS, partial 18S rRNA, *coxII-*16S mtDNA and *coxI* gene sequences of different *Meloidogyne* spp. from GenBank were used for phylogenetic reconstruction. Outgroup taxa for each dataset were chosen according to previous published data [16, 37]. Multiple alignments of the different genes were made using the Q-INS-i algorithm of MAFFT v. 7.205 [38], strategy FFT-NS-1 with default parameters. Sequence alignments were visualized using BioEdit [39] and edited by Gblocks v0.91b [40] in Castresana Lab server (http://molevol.cmima.csic.es/castresana/Gblocks_server.html) using the less stringent option (Minimum number of sequences for a conserved or a flanking position: 50% of the number of sequences + 1; maximum number of contiguous non-conserved positions: 8; minimum length of a block: 5; allowed gap positions: with half). The sequences of *M*. *oleae* sp. nov. were compared with GenBank nematode sequences using the BLASTn homology search program at NCBI database (https://blast.ncbi.nlm.nih.gov/Blast.cgi?PAGE_TYPE=BlastSearch). Phylogenetic analyses of the sequence data sets were performed based on Bayesian inference (BI) using MrBayes 3.1.2 [41]. The best fitted model of DNA evolution was obtained using jModelTest v. 2.1.7 [42] with the Akaike Information Criterion (AIC). The Akaike-supported model, the base frequency, the proportion of invariable sites, and the gamma distribution shape parameters and substitution rates in the AIC were then used in phylogenetic analyses. BI analyses were performed under TIM3+I+G (namely, transition model with a proportion of invariable sites and a gamma-shaped distribution) model for D2-D3 expansion segments of 28S rRNA, a TIM2+I+G model for ITS rRNA, a GTR+I+G (namely, general time reversible with a proportion of invariable sites and a gamma-shaped distribution) model for the partial 18S rDNA, TVM+I+G model for *cox*II-16S mtDNA, and finally GTR+G for the partial *cox*I region. These BI analyses were run separately per dataset using four chains for 2 × 10^6^ generations. The Markov chains were sampled at intervals of 100 generations. Two runs were performed for each analysis. After discarding burn-in samples and evaluating convergence, the remaining samples were retained for further analyses. The topologies were used to generate a 50% majority rule consensus tree. Posterior probabilities (PP) are given on appropriate clades. Trees were visualised using FigTree software V.1.42 (http://tree.bio.ed.ac.uk/software/figtree/).

### Nomenclatural acts

The electronic edition of this article conforms to the requirements of the amended International Code of Zoological Nomenclature (ICZN), and hence the new name contained herein is available under that Code from the electronic edition. This published work and the nomenclatural acts it contains have been registered in ZooBank, the online registration system for the ICZN. The ZooBank LSIDs (Life Science Identifiers) can be resolved and the associated information viewed through any standard web browser by appending the LSID to the prefix "http://zoobank.org/". The LSID for this publication is: urn:lsid:zoobank.org:pub:0C8D30A6-3562-4283-ACF9-A3F3CE7E03F3. The electronic edition of this work was published in a journal with an ISSN, and has been archived and is available from the following digital repositories: PubMed Central, LOCKSS.

### Explanatory variables data from olive orchards

Deterministic processes explaining the heterogeneity of RKNs infesting soils from cultivated olive were compiled in four sets of explanatory variables related with climate, soil, topography, or agronomic management practices (Table 2). Since no agronomic management practices are applied in wild olives, these sampling points were not included in the analysis. The climate data set comprised 21 abiotic broad range variables including bioclimatic predictors (BIOCLIM; [43]), annual mean standardized drought index (DI), and annual intensity of rainfall deficit (RD) [44].

**Table 2:** Explanatory variables sets used to assess community composition of *Meloidogyne* spp. in cultivated olives in Andalusia, southern Spain.

Variable	Details	Variables	Details
**Climatic**		**Topography**	
BIO1-BIO19	*Bioclimatic predictors*	Elevation	*Average elevation of olive orchard*
DI	*Cumulative monthly rainfall anomalies*	Slope	*Average slope of olive orchard*
RD	*Mean Annual Intensity of rainfall deficit*	SWI	*Saga Wetness Index*
		AC	*Altitude above channels*
**Soil**		DN	*Distance to river network*
CEC	*Cation Exchange Capacity*	Convexity	*Terrain convexity*
Ca	*Calcium content*	Aspect	*Terrain aspect*
Mg	*Magnesium content*		
Na	*Sodium content*	**Agronomic management**	
Kech	*Exchangeable Potassium*	Cultivar	*Cultivar olive tree growing on olive orchard*
CO3	*Carbonate content*	Age	*Age of olive orchard*
Pext	*Extractable Phosphorus*	Density	*Density plantation on olive orchard (traditional*, *intensive and high-density)*
SOM	*Soil Organic Matter*	Irrigation	*Irrigation regimen on olive orchard (e*.*g*. *rainfed or irrigated)*
Corg	*Total Carbon content*	Water	*Source of irrigation water (e*.*g*. *underground or superficial water source)*
Norg	*Total Nitrogen content*	Canopy	*Management practices below olive tree canopy on olive orchard (e*.*g*. *herbicide application / tilling or not management practices)*
C:N	*Carbon/Nitrogen ratio*	Alley	*Management practices on alley of olive orchard (e*.*g*. *tilling*, *herbicide application*, *grinding or vegetative cover)*
pH (KCl)	*Soil pH*	Alley Cover	*Any kind of vegetative cover on alley of olive orchard (e*.*g*. *natural or grasses vegetation)*
Clay	*Clay content*		
Sand	*Sand content*		
Silt	*Silt content*		

Soil data comprised 12 parameters related with physicochemical characteristics including cation exchange capacity (CEC), Ca, Mg, exchangeable K, Na, carbonate content (CO3), extractable P, soil organic matter (SOM), total organic carbon (Corg) and nitrogen (Norg), C:N ratio, and pH (KCl). In addition, soil texture was also estimated by the relative amounts of sand, clay and silt according to soil texture Bouyoucos method [45].

A third set of variables included topography variation of the olive growing area. We calculated values for seven topographic predictors: elevation, slope, topographic wetness index (SWI), altitude above water channels (AC), and distance to river networks (DN), terrain convexity and aspect. Overall, topography variation has been described as a good indicator for soil heterogeneity (i.e. aspect describes differences in a light availability) [46, 47]. Since soil nematode activity is influenced by soil moisture [48], we included the two indices SWI and AC which are commonly used to quantify topographical control on hydrological processes [47]. We also included the euclidean distance between olive orchard and river network which may be used to quantify the potential mechanisms of organism dispersal controlled by their habitat and topographic features [49]. Each of these variables was derived by a digital elevation model (DEM) at 5 m ground solution [50]. Slope, aspect and terrain convexity were calculated from DEM according to second-degree polynomial adjustment method [51] using the library morphometry of the open source GIS SAGA [52]. SWI and AC were computed from DEM according to procedures described by Boehner and Selige [53] using the hydrology and terrain analysis modules in GIS SAGA. From each commercial olive orchard, topographic variables were defined as the mean of the values derived in each olive tree randomly selected for sampling soil.

Finally, the fourth set of variables encompassed characteristics related to agronomic management practices (Table 2). For the first subset, we used age of olive plantation and olive cultivars. The age of olive orchard was provided by the landowner ranging from about 6 to 100 years which belonged to 7 olive cultivars The second subset comprised 7 explanatory variables: olive plant density, irrigation regimen and source of irrigation water in olive orchards, agronomic practices below olive tree canopy and on alley, and type of vegetation cover on alley of olive orchards. Data on plant density was categorized into three classes (e.g. traditional, intensive and super high-density olive orchards) as suggested by Rallo *et al*. [54].

### Statistical analyses

Data analyses were accomplished in two steps that included: (i) fit models to each explanatory variables data set, and (ii) a canonical redundancy ordination analysis (RDA) among community composition and explanatory variables significantly structuring community patterns of RKNs. All statistical analyses were performed using R v.3.3.0 freeware [55].

We used a prevalence matrix data of RKNs that was Hellinger-transformed prior to the analysis [56]. An accurate data exploration was carried out for each variable set. All numeric variables were tested for normality (Shapiro-Wilk test) and homogeneity of variance (Fligner test). Then, soil physicochemical variables were standardized; and soil texture was categorized in 12 texture classes according to the USDA soil classification in order to avoid collinearity among texture variables [57]. Likewise, we used the third degree polynomial function of five topographic variables (i.e. elevation, slope, SWI, altitude above channel and convexity) following the recommendation by Legendre *et al*. [58]. Since aspect is a circular variable measured in degrees, it was transformed in order to make it linear using the cos(aspect) and sin(aspect). We therefore obtained 18 reconstructed variables from the seven original topographic variables. In addition, numeric variables within each data set were tested for collinearity based on the variance inflation factor (VIF) method [59]. Then, numeric covariates with a VIF value > 3 were iteratively excluded to minimize collinearity effects using the corvif function in the AED package [60].

A forward selection procedure was performed to fit the explanatory variables significantly structuring the community composition for each variables data set. Categorical predictors were transformed as dummy variables prior to forward selection analysis. We used a modified forward selection method based on a permutation procedure with two stopping criteria using 9999 random permutations as suggested by Blanchet *et al*. [61]. This analysis was carried out using the packfor package [62]. Finally, a redundancy ordination analysis (RDA) was performed between the fitted variables and community composition based on prevalence data in order to interpret the relationships between environmental variables and RKNs species. In addition, we applied a trend-surface analysis on multivariate data by means of RDA with the aim to partitioning spatial effects of environmental variables on presence/absence data of RKNs. We obtained a set of independent spatial models related to each canonical axis. The canonical analyses were computed using the packages vegan [63] and ade4 [62].

## Results

### Taxon sampling, abundance and prevalence of *Meloidogyne* spp. in cultivated and wild olive

All *Meloidogyne* spp. found in this study, including specimens of sampling sites used in morphological and/or molecular analyses, are shown in Table 1 and Fig 1. The overall presence of *Meloidogyne* spp. in the olive plants representing Southern Spain was 6.6%, with 6.6% and 6.5% for cultivated and wild olive, respectively. Overall, six species were found infecting cultivated or wild olive trees. *Meloidogyne baetica* was highly specific to wild olive and it has been not found outside its description area (Vejer, Cádiz). On the other hand, *M*. *arenaria* and *M*. *incognita* were found only in cultivated olive, whereas the rest of species, *M*. *oleae* sp. nov., *M*. *hapla* and *M*. *javanica* could be found in both types of olive trees. Soil nematode population density (number of specimens) and prevalence per species in wild and cultivated olives is shown for soil (Table 3) and root samples (Table 4). The most prevalent nematode for both types of olives was *M*. *javanica*, with 4.0% of the sampled points infested, and followed by *M*. *arenaria* (0.8%) and *M*. *incognita* (0.8%). The highest nematode densities in wild olive were found for *M*. *oleae* sp. nov. with 121 nematodes per 500 cm^3^ of soil, while for cultivated olive was *M*. *javanica* with 10,137 nematodes per 500 cm^3^ of soil, followed by *M*. *incognita* with 5,727 nematodes per 500 cm^3^ of soil. These identified species showed no differences with previously described isozyme patterns and no variability was found in their identification using the different methods described in material and methods. These densities in soil were not correlated with the densities of nematodes in olive roots for the most damaging species, in which case the major densities were found for *M*. *incognita* (859.9 eggs+J2s/g of root), followed by *M*. *javanica* (323.1 eggs+J2s/g of root) and *M*. *oleae* sp. nov. (105.4 eggs+J2s/g of root) (Table 4).

**Table 3:** Soil nematode population density (number of specimens) per 500 cm^3^ of soil and prevalence (%) of *Meloidogyne* spp. in soil in wild and cultivated olives in Andalusia, southern Spain.

Host plant ^a^	Wild olive (W)				Cultivated olive (C)					Global data (W + C)			
Number of samples	123				376					499			
	Average^b^	Min^b^	Max^b^	Prevalence^c^	Average	Min		Max	Prevalence	Average	Min	Max	Prevalence
***Meloidogyne* spp.**													
*Meloidogyne arenaria*	-	-	-	-	45.3	2		138	1.064	45.3	2	138	0.802
*Meloidogyne baetica*	11.0	8	14	1.626	-	-		-	-	11.0	8	14	0.401
*Meloidogyne hapla*	9.0	9	9	0.813	2.0	2		2	0.266	5.5	2	9	0.401
*Meloidogyne incognita*	-	-	-	-	1,951.7	4		5,727	1.064	1,464.8	4	5,727	0.802
*Meloidogyne javanica*	19.4	1	49	4.065	733.2	1		10,137	3.989	554.8	1	10,000	4.008
***Meloidogyne oleae* sp. nov.**	121.0	121	121	0.813	25.0	4		46	0.532	57.0	4	121	0.601

^a^ Host plant: W = wild olive; C = cultivated olive.

^b^ Population average was calculated as the mean of *Meloidogyne* nematodes per 500 cm^3^ of soil in all the infested sampling points for these species.

^c^ The prevalence was computed by dividing the numbers of samples in which the *Meloidogyne* species was observed by the total number of samples and expressed as a percentage

(-) not found

**Table 4:** Nematode population density (number of specimens) and prevalence (%) of *Meloidogyne* spp. in roots of wild and cultivated olives in Andalusia, southern Spain.

Host plant^a^	Wild olive (W)				Cultivated olive (C)				Global data (W + C)			
Number of samples	123				376				499			
	Average^b^	Min^b^	Max^b^	Prevalence^c^	Average	Min	Max	Prevalence	Average	Min	Max	Prevalence
***Meloidogyne* spp.**												
*Meloidogyne arenaria*	-	-	-	-	6.1	0.8	17.6	1.064	6.1	0.8	17.7	0.802
*Meloidogyne baetica*	5.2	1.1	9.4	1.626	-	-	-	-	5.2	1.1	9.4	0.401
*Meloidogyne hapla*	0.6	0.61	0.61	0.813	0.4	0.4	0.4	0.266	0.5	0.4	0.6	0.401
*Meloidogyne incognita*	-	-	-	-	229.1	8.2	859.9	1.064	229.2	8.2	859.9	0.802
*Meloidogyne javanica*	20.3	0.7	83.6	4.065	42.2	1.3	323.1	3.989	36.7	0.7	323.1	4.008
***Meloidogyne oleae* sp. nov.**	19.9	19.9	19.9	0.813	53.1	0.7	105.4	0.532	42.2	0.7	105.4	0.601

^a^ Host plant: W = wild olive; C = cultivated olive.

^b^ Population average was calculated as the mean of *Meloidogyne* nematodes (eggs+ J2) per gram of olive fresh root in all the infested sampling points for these species.

^c^ The prevalence was computed by dividing the numbers of samples in which the *Meloidogyne* species was observed by the total number of samples and expressed as a percentage

(-) not found

### Taxonomic treatment

Nematoda Linnaeus, 1758

Tylenchida Thorne, 1949

Meloidogynidae Skarbilovich, 1959

Meloidogyninae Skarbilovich, 1959

*Meloidogyne* Göeldi, 1892

***Meloidogyne oleae* Archidona-Yuste, Cantalapiedra-Navarrete, Liébanas, Rapoport, Castillo & Palomares-Rius, sp. nov.** *urn*:*lsid*:*zoobank*.*org*:*act*:*80A4D086-0EBF-468D-B881-70B373E774A7*

Figs 2–10; Tables 5 and 6

**Fig 2:**
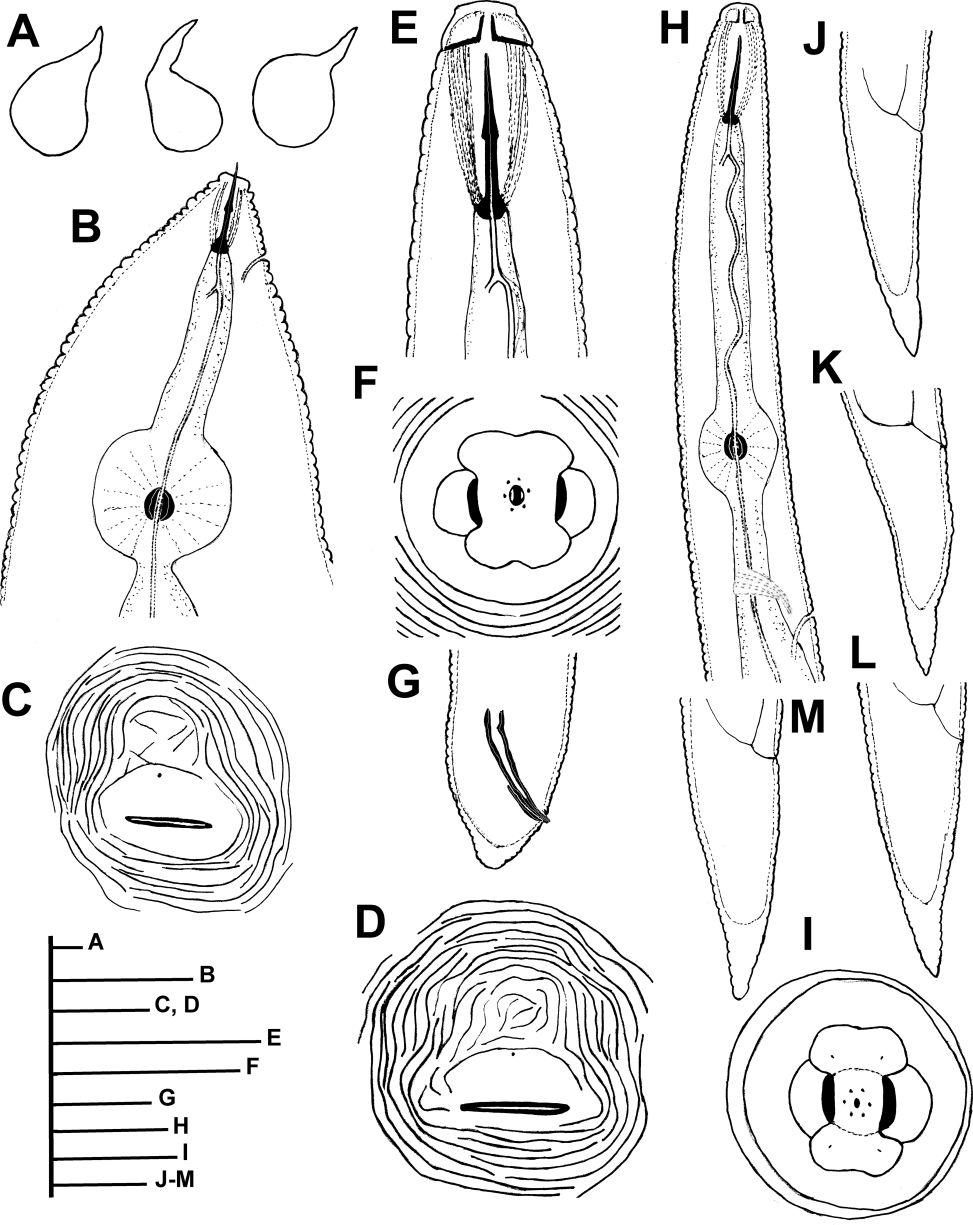
Line drawings of *Meloidogyne oleae* sp. nov., female, male and second-stage juvenile paratypes. A) Entire body of female. B) Anterior region of female. C, D) Perineal pattern. E) Anterior region of male. F) *En face* view of male lip region. G) Male tail. H) Pharyngeal region of second-stage juvenile. I) *En face* view of second-stage juvenile lip region. J-M) Second-stage juvenile tails. Scale bars: A = 200 μm; B-E, G, H = 20 μm; F = 5 μm; I = 2 μm; J-M = 10 μm.

**Fig 3:**
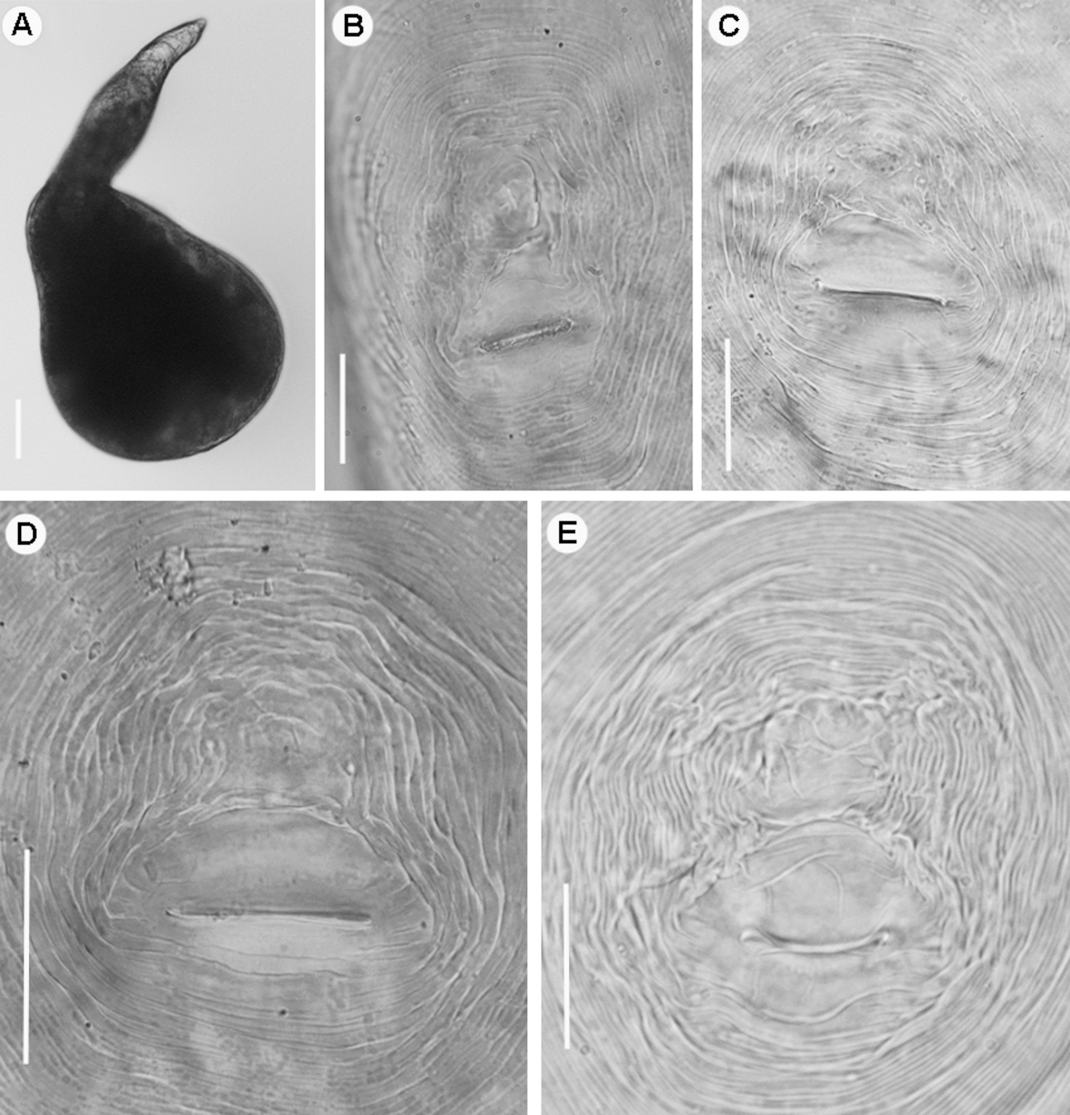
Light micrographs of *Meloidogyne oleae* sp. nov., female paratypes. A) Whole female. B–E) Detail of female perineal patterns. Scale bars: A = 100 μm; B-E = 20 μm.

**Fig 4:**
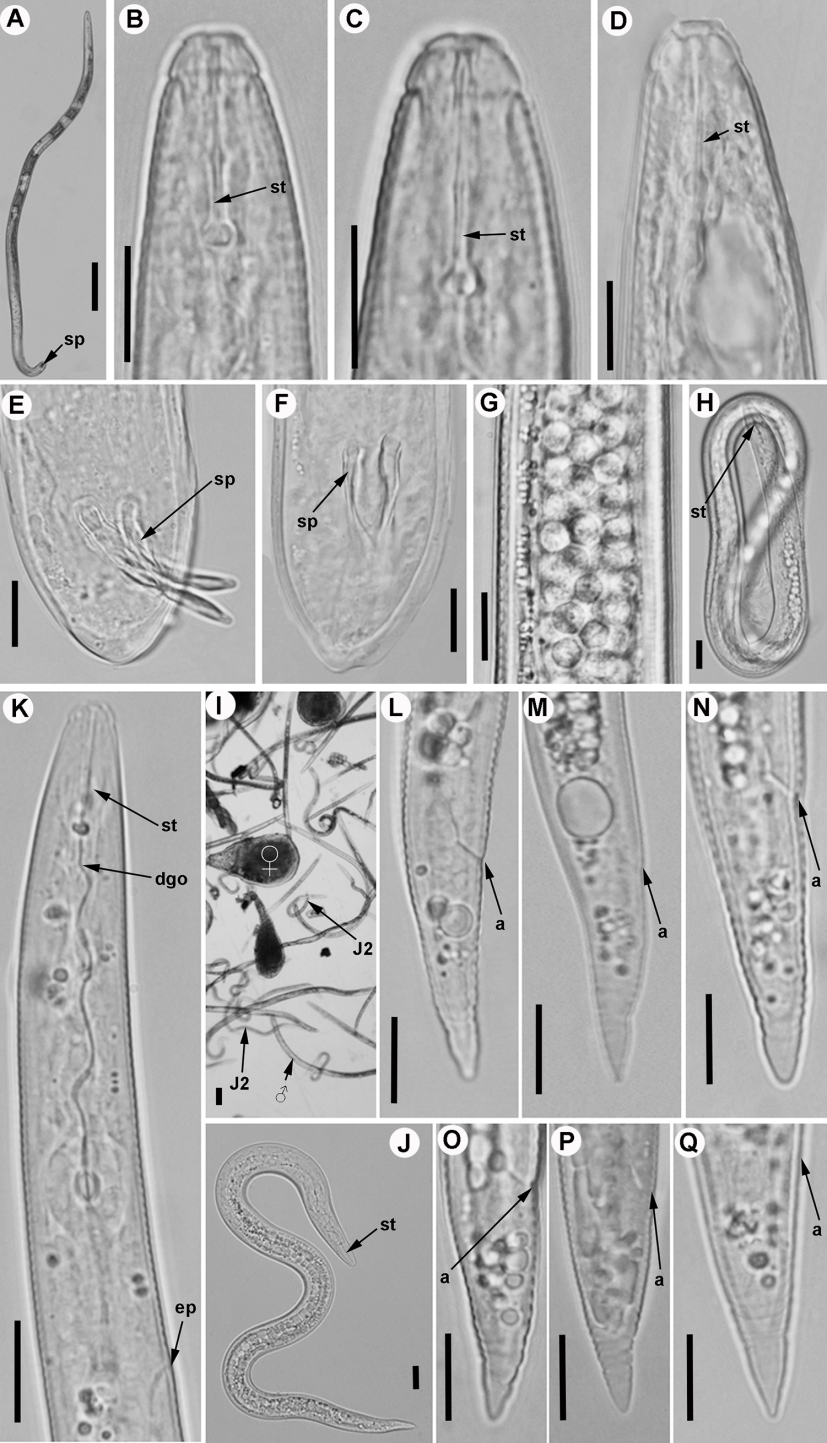
Light micrographs of *Meloidogyne oleae* sp. nov., male and second-stage juvenile paratypes. A) Whole male. B-D) Male lip region. E-F) Male tail showing spicules. G) Detail of sperm cells. H) Detail of embryonated egg showing stylet of second-stage juvenile. I) All life stages. J) Whole second-stage juvenile. K) Anterior region of second-stage juvenile showing excretory pore, stylet and dorsal gland orifice. L–Q) Tail of second-stage juveniles (J2). Abbreviations: a = anus; dgo = dorsal gland orifice; = ep = excretory pore; sp = spicules; st = stylet. Scale bars: A, I = 100 μm; B-H, J-Q = 10 μm.

**Fig 5:**
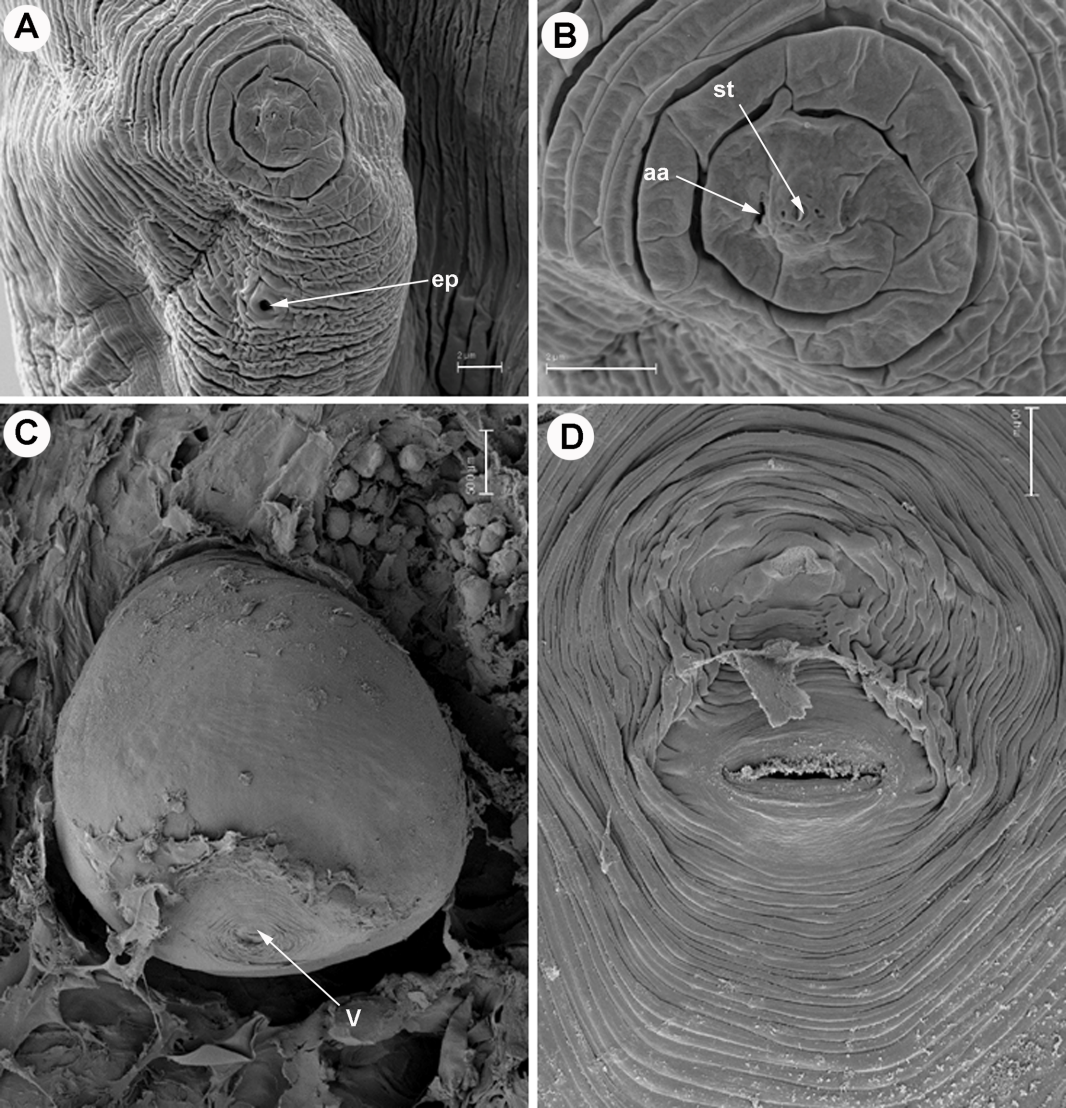
SEM micrographs of *Meloidogyne oleae* sp. nov., female paratypes. A, B) Female lip region showing position of excretory pore and amphidial aperture. C) Whole female included in wild olive root. D) Detail of female perineal pattern. Abbreviations: aa = amphidial aperture; ep = excretory pore; st = stylet; V = vulva. Scale bars A, B = 2 μm; C = 500 μm; D = 10 μm.

**Fig 6:**
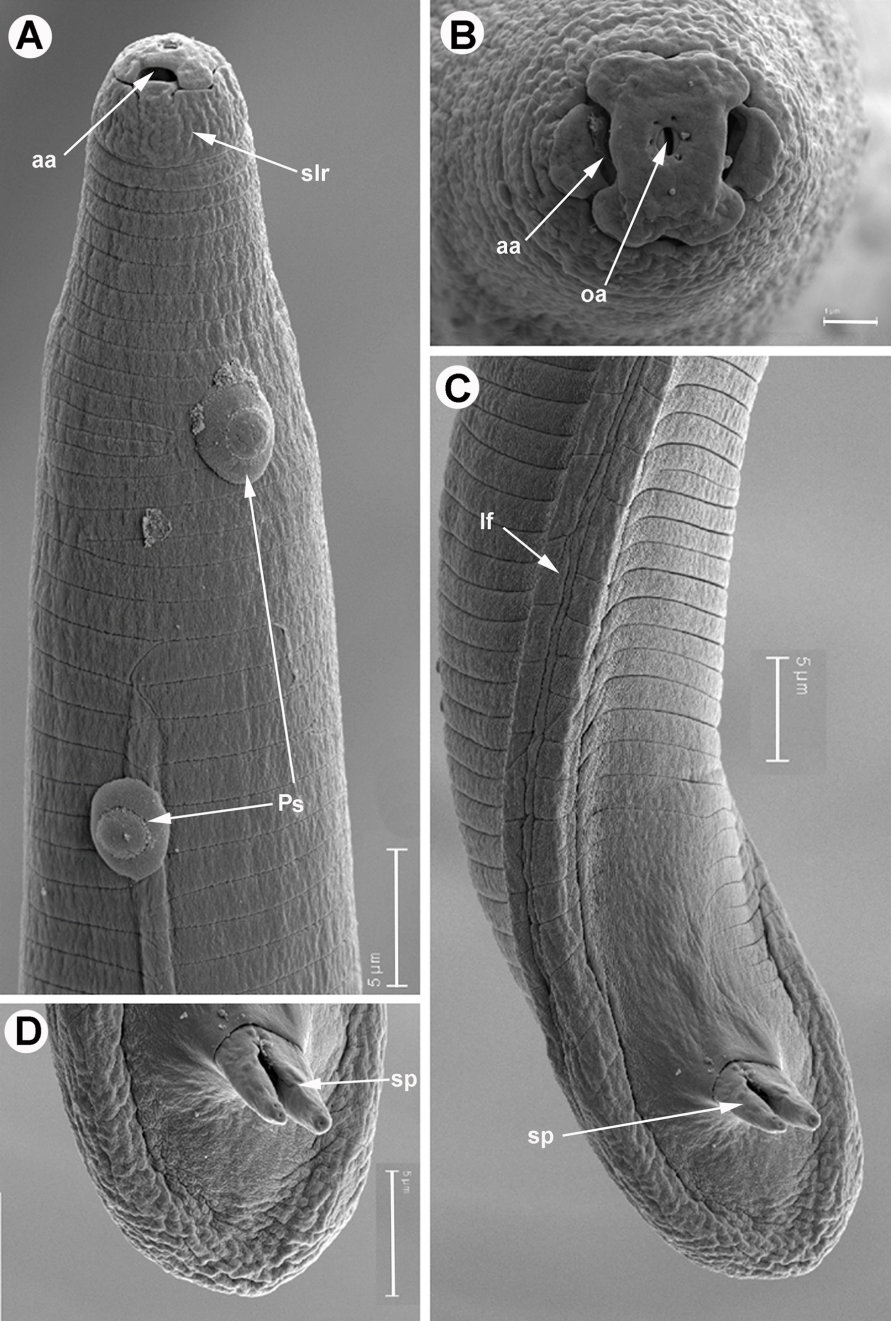
SEM micrographs of *Meloidogyne oleae* sp. nov., male paratypes. A) Anterior region showing smooth lip region, amphidial aperture, and endospore bacterial of *Pasteuria* sp. B) *En face* view showing amphidial and oral apertures. C, D) Detail of tail showing lateral field and spicules. Abbreviations: aa = amphidial aperture; oa = oral aperture; lf = lateral field; Ps = endospore *Pasteuria* sp.; slr = smooth lip region. Scale bars A, C, D = 5 μm; B = 1 μm.

**Fig 7:**
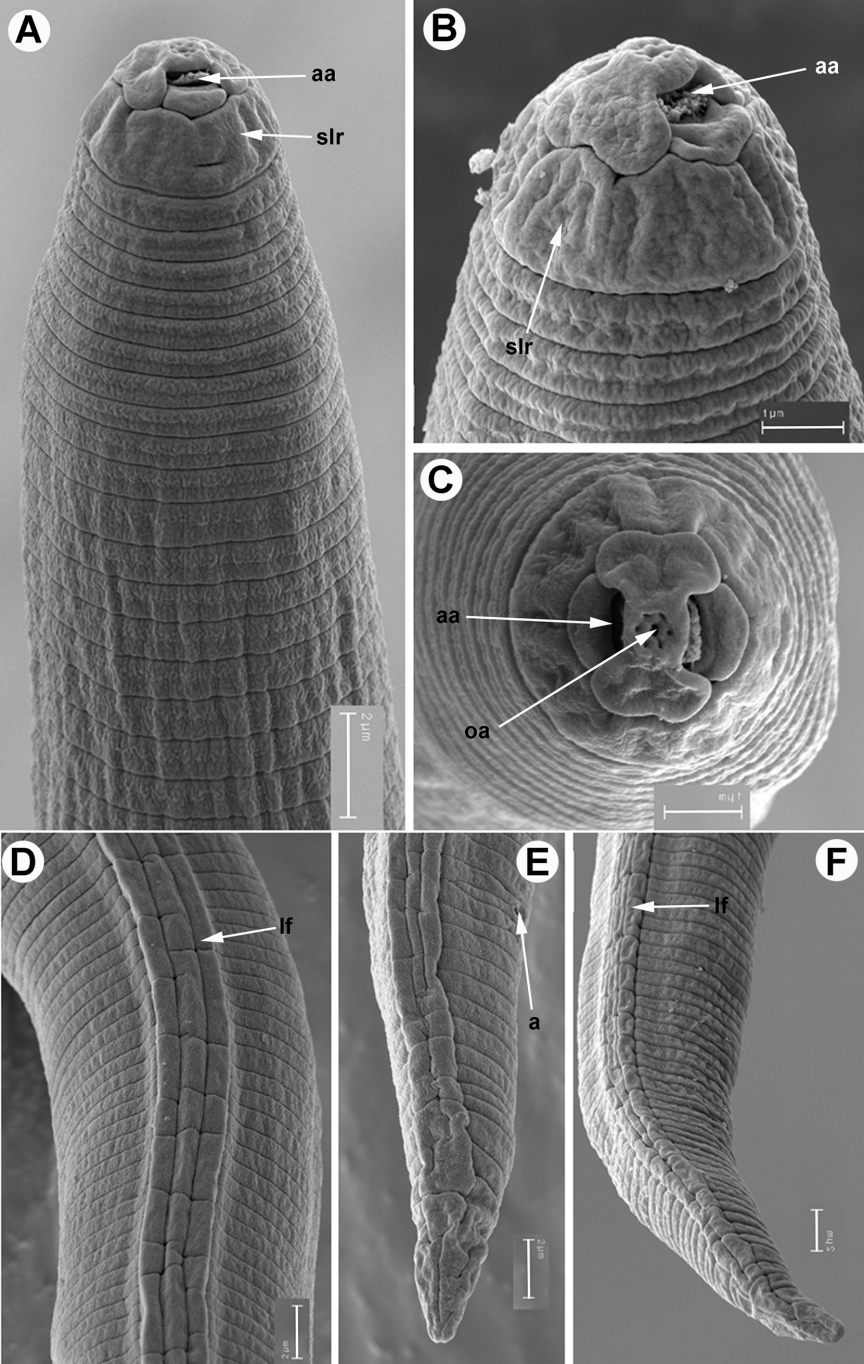
SEM micrographs of *Meloidogyne oleae* sp. nov., second-stage juvenile paratypes. A, B) Smooth lip region and amphidial aperture. C) *En face* view of lip region showing amphidial and oral apertures. D) Detail of lateral field at mid-body. E-F) Tail. Abbreviations: a = anus; aa = amphidial aperture; oa = oral aperture; lf = lateral field; slr = smooth lip region. Scale bars A, D-F = 2 μm; B, C = 1 μm.

**Fig 8:**
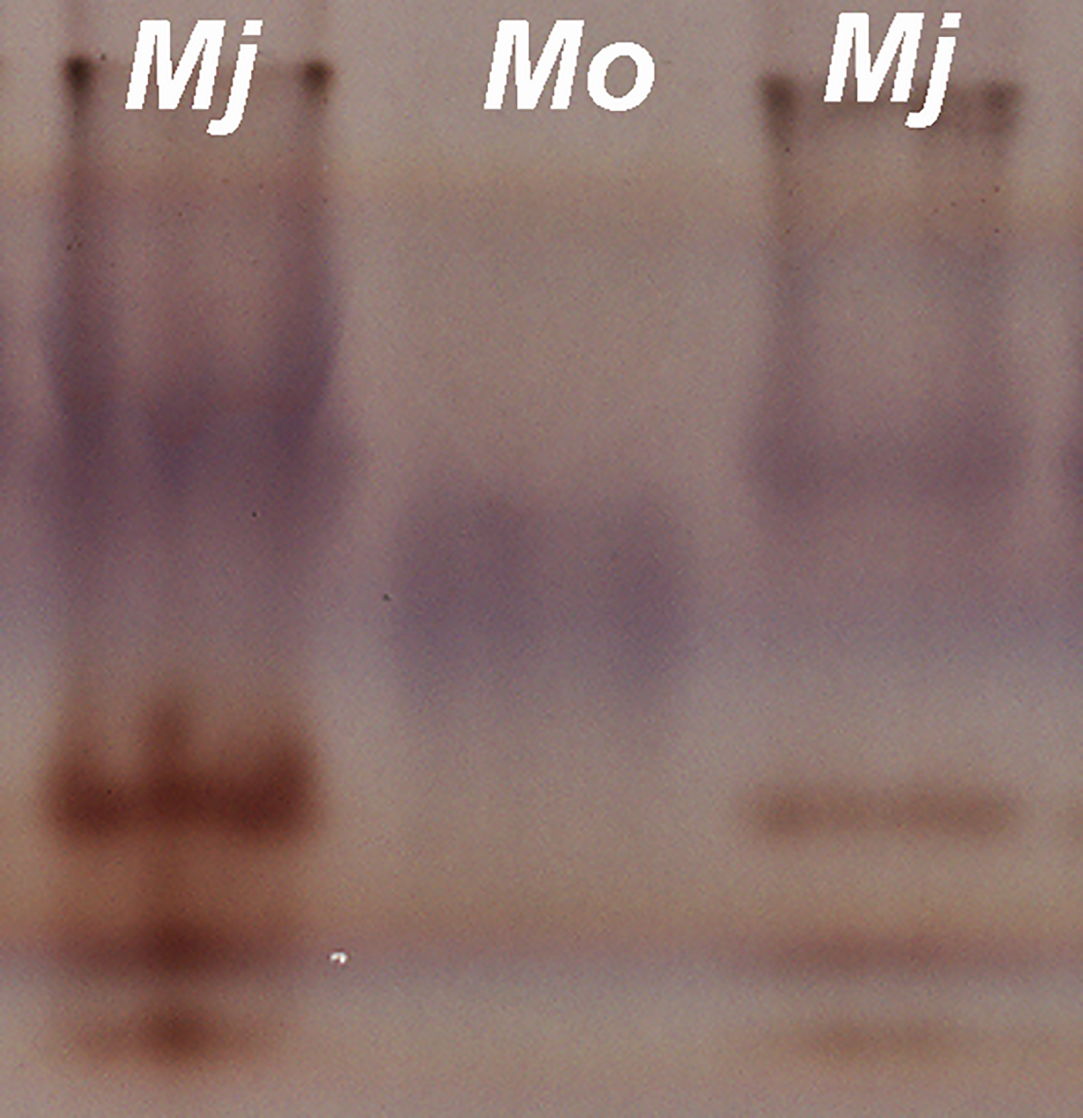
Esterase (Est) (in black) and malate dehydrogenase (Mdh) (in purple) electrophoresis patterns of protein homogenates from five young, egg-laying females of *Meloidogyne oleae* n. sp. nov., and five young, egg-laying females of *M*. *javanica* (reference population).

**Fig 9:**
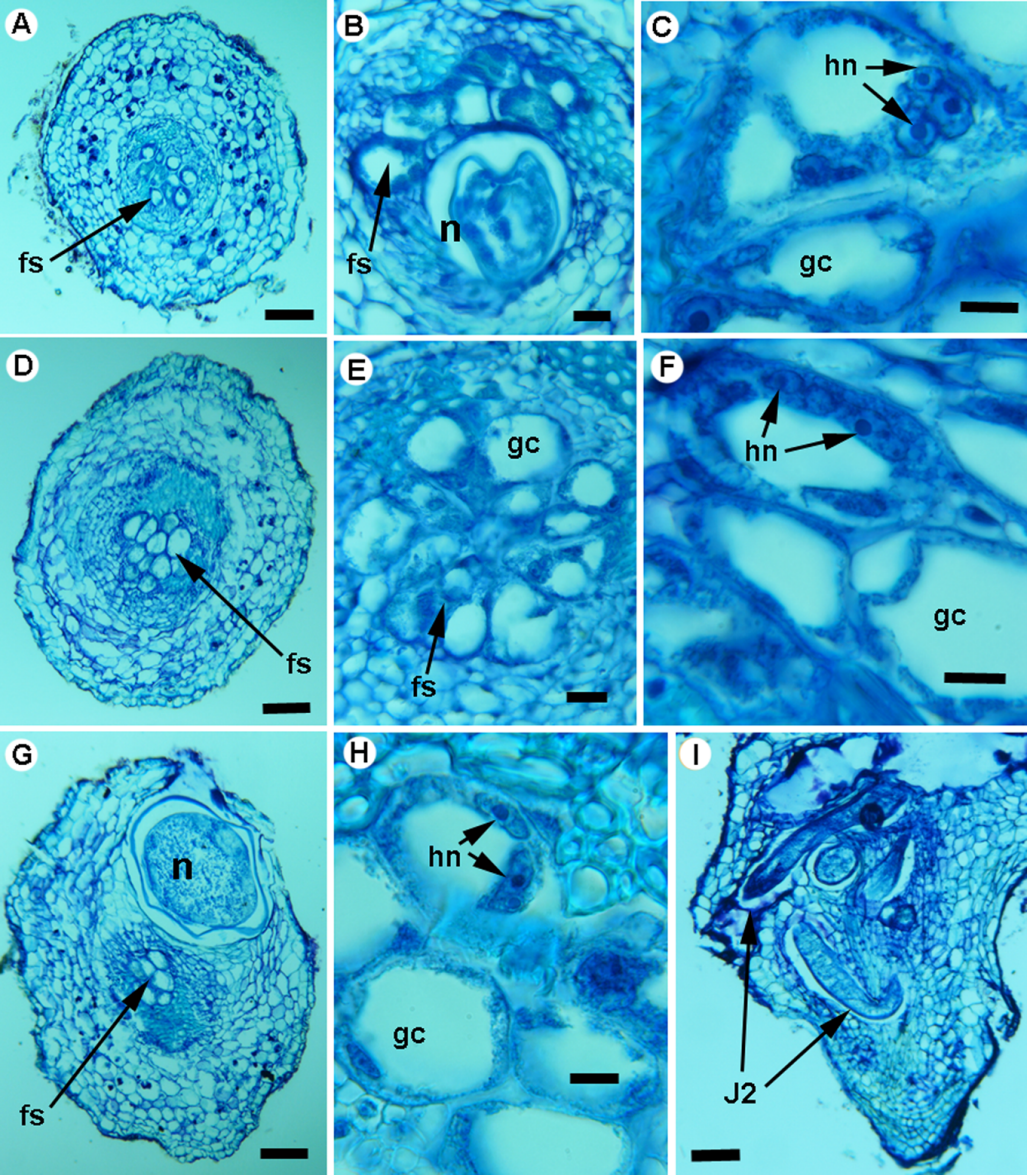
**Histological sections of *Meloidogyne oleae* sp. nov. on wild (A-F) and cultivated (G-I) olives.** A, D, G. Transverse section of young olive roots showing permanent feeding sites in vascular parenchyma. I. Apical longitudinal section showing initial infections by second-stage juveniles or J2 (arrow). B, C, E, F, H. Detailed images of feeding sites showing multinucleate giant cells with hypertrophied nuclei and nucleoli. Staining: toluidine blue O. Abbreviations: fs = feeding site; gc = giant cell; hn = hypertrophied nuclei; J2 = second-stage juvenile; n = nuclei. Scale bars = A, B, D, E, G, I = 100 μm; C, F, H = 10 μm.

**Fig 10:**
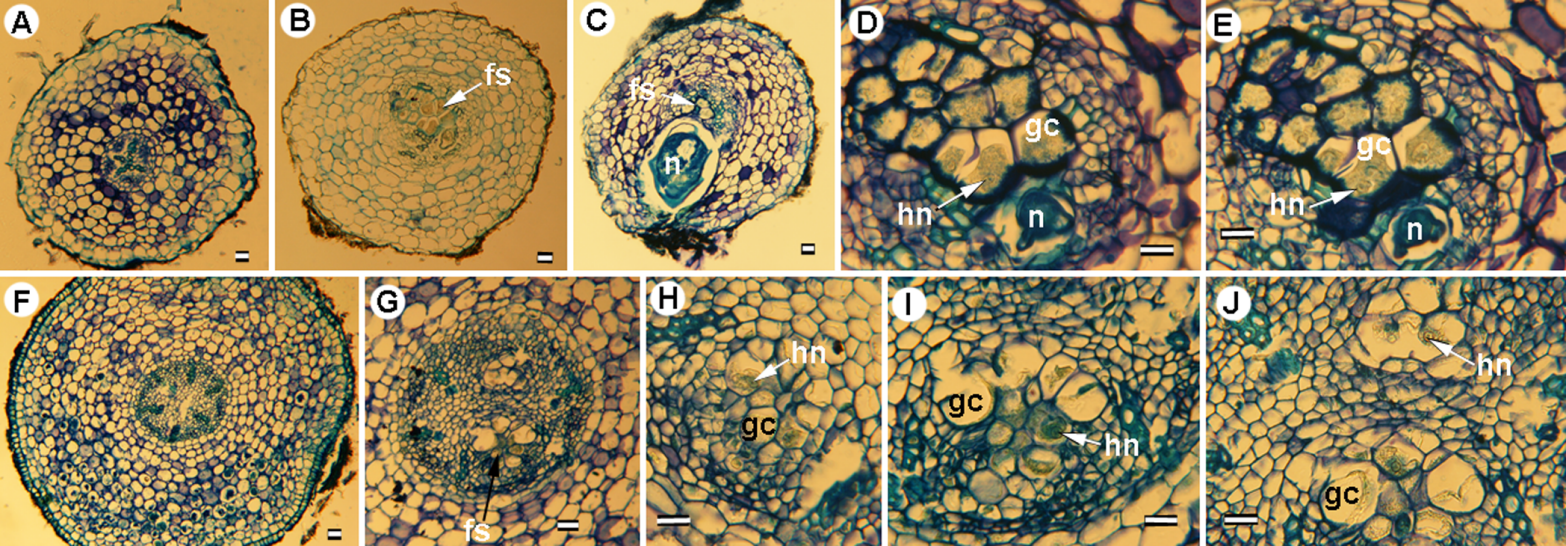
**Histological sections of *Meloidogyne oleae* sp. nov. on lesser periwinkle (*Vinca minor* L.) (A-E) and rosebush (*Rosa* sp.) (F-J).** A, F. Transverse sections of healthy lesser periwinkle and rosebush roots. B, C. G. Transverse sections of young lesser periwinkle and rosebush roots showing permanent feeding sites in vascular parenchyma. D, E, H, I, J. Detailed images of feeding sites showing multinucleate giant cells with hypertrophied nuclei and nucleoli. Staining: toluidine blue O. Abbreviations: fs = feeding site; gc = giant cell; hn = hypertrophied nuclei; n = nuclei. Scale bars = 100 μm.

**Table 5:** Morphometrics of females, males and second-stage juveniles of *Meloidogyne oleae* sp. nov. from the rhizosphere of wild olive at Tolox (Málaga province) southern Spain^a^.

Characters/ratio^b^	Holotype	Paratype females	Paratype males	Paratype J2
**n**		19	20	16
**L**	701	591 ± 85.9	1237 ± 238	371 ± 12.1
		(445–790)	(838–1762)	(351–385)
**Maximum body diam.**	418	356 ± 54.1	25.8 ± 5.4	14.3 ± 0.8
		(271–421)	(17.5–38.5)	(13.0–16.0)
**Stylet length**	13.0	13.3 ± 0.5	15.7 ± 1.1	12.3 ± 0.6
		(12.5–14.0)	(13.5–18.0)	(11.0–13.0)
**DGO**	4.5	4.4 ± 0.7	5.1 ± 0.7	3.1 ± 0.5
		(3.0–5.5)	(4.0–6.0)	(2.5–3.5)
**Metacorpus length**	32.0	31.0 ± 5.5	15.4 ± 2.9	10.6 ± 1.2
		(23.0–39.0)	(13.5–20.5)	(8.5–12.5)
**Metacorpus width**	28.0	27.1 ± 6.0	10.1 ± 1.9	7.0 ± 0.9
		(20.0–38.0)	(8.0–12.5)	(6.0–8.5)
**Stylet knob width**	3.0	3.0 ± 0.4	2.5 ± 0.3	2.1 ± 0.5
		(2.5–3.5)	(2.0–3.0)	(1.5–2.5)
**Lip region width**	8.0	7.8 ± 0.4	7.6 ± 0.6	5.4 ± 0.5
		(7.0–8.0)	(6.5–8.5)	(4.5–6.0)
**Anterior end to excretory pore**	13.0	12.7 ± 0.5	118.3 ± 9.2	68.0 ± 4.2
		(11.5–13.5)	(79.0–140.5)	(57.0–75.5)
**Excretory pore/stylet length**	1.0	1.0 ± 0.05	-	-
		(0.9–1.0)	-	-
**Vulva length**	22.0	19.5 ± 1.7	-	-
		(18.0–22.0)	-	-
**Distance from vulva to anus**	24.0	23.3 ± 1.0	-	-
		(22.0–24.0)	-	-
**Anal body diameter**	-	-	14.4 ± 1.9	9.3 ± 0.9
		-	(12.0–17.0)	(8.0–11.0)
**Tail length**	-	-	9.5 ± 1.7	25.1 ± 1.6
		-	(8.0–12.5)	(23.0–28.5)
**Hyaline tail region**	-	-	-	7.4 ± 1.2
		-	-	(6.5–11.0)
**Spicules length**	-	-	26.7 ± 3.0	-
		-	(21.0–32.0)	-
**Gubernaculum**	-	-	8.3 ± 1.4	-
		-	(6.5–11.5)	-
**a**	1.7	1.7 ± 0.2	48.6 ± 9.6	26.1 ± 1.5
		(1.4–2.1)	(27.3–64.1)	(24.0–29.0)
**b**	-	-	10.0 ± 2.1	5.3 ± 0.4
		-	(7.8–14.9)	(4.8–6.1)
**c**	-	-	134.4 ± 24.2	14.8 ± 0.8
		-	(107.0–178.1)	(13.5–16.0)
**c´**	-	-	0.7 ± 0.1	2.7 ± 0.2
		-	(0.6–1.0)	(2.4–3.2)

^a^ Measurements are in μm and in the form: mean ± standard deviation (range).

^b^ Abbreviations as defined in Siddiqi [26].

**Table 6:** Morphometrics of males and second-stage juveniles of *Meloidogyne oleae* sp. nov. from the rhizosphere of cultivated olive at Antequera (Málaga province) southern Spain^a^ in sampling point JAO28 and JAO31.

	JAO28		JAO31	
Characters/ratio^b^	males	J2	males	J2
**n**	5	6	8	6
**L**	1056 ± 88	369 ± 11	1179 ± 162	391 ± 28.8
	(995–1210)	(355–382)	(1024–1522)	(360–437)
**Maximum body diam.**	20.1 ± 1.7	13.0 ± 0.9	23.1 ± 2.9	14.2 ± 0.5
	(18.0–22.5)	(12.0–14.0)	(17.5–26.0)	(13.5–15.0)
**Stylet length**	14.7 ± 1.0	11.4 ± 0.6	14.3 ± 1.2	11.3 ± 0.3
	(13.5–16.0)	(10.5–12.0)	(12.5–15.5)	(11.0–11.5)
**DGO**	4.3 ± 0.3	2.9 ± 0.4	4.6 ± 0.3	4.3 ± 0.4
	(4.0–4.5)	(2.5–3.5)	(4.5–5.0)	(4.0–4.5)
**Metacorpus length**	17.7 ± 1.5	7.7 ± 0.3	17.5 ± 0.7	7.0 ± 0.7
	(16.0–19.0)	(7.5–8.0)	(17.0–18.0)	(6.5–7.5)
**Metacorpus width**	8.2 ± 1.6	10.5 ± 1.0	12.5 ± 0.7	10.8 ± 0.4
	(7.0–10.0)	(9.5–12.5)	(12.0–13.0)	(10.5–11.0)
**Stylet knob width**	2.1 ± 0.3	1.9 ± 0.3	2.7 ± 0.3	1.8 ± 0.3
	(2.0–2.5)	(1.5–2.0)	(2.5–3.0)	(1.5–2.0)
**Lip region width**	7.8 ± 0.3	4.8 ± 0.3	7.9 ± 0.3	5.1 ± 0.2
	(7.5–8.5)	(4.5–5.0)	(7.5–8.5)	(5.0–5.5)
**Anterior end to excretory pore**	97.4 ± 20.9	74.6 ± 4.4	116.7 ± 8.5	71.6 ± 2.5
	(74–115)	(70.5–80.0)	(108.0–127.5)	(69.0–75.5)
**Anal body diameter**	11.3 ± 1.0	8.7 ± 0.7	13.7 ± 3.6	7.9 ± 0.6
	(10.5–12.5)	(7.5–9.5)	(10.5–18.0)	(7.0–8.5)
**Tail length**	7.3 ± 1.0	23.0 ± 2.0	9.5 ± 2.8	21.8 ± 1.4
	(6.0–8.5)	(21.0–26.0)	(7.0–13.0)	(19.5–23.5)
**Hyaline tail region**	-	6.5 ± 0.7	-	6.0 ± 0.9
	-	(5.5–7.5)	-	(5.0–7.0)
**Spicules length**	26.4 ± 1.9	-	26.6 ± 3.8	-
	(24.5–28.5)	-	(21.0–29.0)	-
**Gubernaculum**	7.6 ± 0.6	-	7.0 ± 0.5	-
	(7.0–8.5)	-	(6.5–7.5)	-
**a**	52.7 ± 3.4	28.7 ± 1.9	51.4 ± 6.9	27.6 ± 1.9
	(47.4–52.1)	(27.3–31.5)	(43.8–63.2)	(25.7–31.2)
**b**	8.3 ± 1.4	5.8 ± 0.4	11.9 ± 2.0	5.0 ± 0.2
	(7.3–9.3)	(5.5–6.3)	(10.0–14.9)	(4.8–5.2)
**T**	47.8 ± 5.0	-	47.5 ± 10.0	-
	(43.5–55.5)	-	(36.4–60.7)	-
**c**	142.9 ± 23.3	16.1 ± 1.2	131.5 ± 19.2	18.0 ± 1.2
	(118.8–175.5)	(14.2–17.6)	(116.2–156.6)	(16.0–19.4)
**c´**	0.6 ± 0.07	2.6 ± 0.2	0.7 ± 0.04	2.7 ± 0.2
	(0.6–0.7)	(2.4–2.9)	(0.6–0.7)	(2.5–3.0)

^a^ Measurements are in μm and in the form: mean ± standard deviation (range).

^b^ Abbreviations as defined in Siddiqi [26].

#### Holotype

Adult female, collected from galled roots of wild olive (*Olea europaea* L. subsp. *europaea* var. *sylvestris*) (36°42'45.0"N, 004°52'39.1"W), at Tolox, Málaga province, Spain; collected by J. Martin Barbarroja and G. Leon Ropero, May 14, 2016; mounted in pure glycerine and deposited in the nematode collection at Institute for Sustainable Agriculture (IAS) of Spanish National Research Council (CSIC), Córdoba, Spain (collection number AR107-2).

#### Paratypes

Adults and juvenile paratypes extracted from root and soil samples collected from the same locality as the holotype; mounted in pure glycerine and deposited in the following nematode collections: Institute for Sustainable Agriculture (IAS) of Spanish National Research Council (CSIC), Córdoba, Spain (collection numbers AR107-5-AR107-9); two juveniles at Istituto per la Protezione Sostenibile delle Piante; and four juveniles at USDA Nematode Collection, Beltsville, MD, USA (T-6974p).

#### Diagnosis

*Meloidogyne oleae* sp. nov. is characterized by a female stylet (12.5–14.0) μm long, a mostly rounded to oval perineal pattern, a moderately low dorsal arch that is mostly rounded, sometimes squarish and generally low, and an excretory pore usually at the level of the anterior end of the procorpus (distance from anterior end to excretory pore (EP)/stylet length (ST) ratio = 0.9–1.0); second-stage juveniles with smooth lip region, tail short belonging to morphospecies Group 2 [64], with a tail terminus tapered with a broad, rounded triangular hyaline area; males with stylet and spicules long, and specific D2-D3, ITS1 rRNA, partial 18S rRNA, *coxI* and partial *coxII-16S* rRNA sequences.

#### Etymology

The species name is derived from the Latin word *oleae* (genitive feminine) = olive (*Olea europaea* subsp. *europaea*), the plants from which the new species was isolated.

#### Description of taxa. Female

Body usually completely embedded in galled tissue, pearly white, globose or pear shaped, with long neck but no posterior protuberance. Lip region continuous with body contour. Labial cap variable in shape, with labial disk and post labial annulus no elevated. In SEM view, the labial disc appears round-squared, slightly raised on the medial and lateral sectors, which are all fused together. Labial framework weakly sclerotized. Stylet short, with an almost straight, rarely curved, cone, cylindrical shaft, and knobs rounded and sloping backwards in most of the specimens. Excretory pore located at the level of stylet knobs, or few body annuli anterior to them. Pharyngeal gland with a large mononucleate dorsal lobe and two sub-ventral gland lobes, usually difficult to see. Perineal pattern mostly rounded-oval (as illustrated in figures), dorsal arch generally low, with fine cuticle striae, which become coarser in the vicinity of perivulval region; lateral fields and punctations not observed. Phasmids distinct, located just above the level of anus. Vulva slit in the middle of the unstriated area (18.0–22.0) μm long, slightly shorter than the vulva-anus distance; anus fold clearly visible, but not always present. Commonly, large egg sac occurs outside the root gall, containing up to 248 eggs.

#### Eggs

Embryonated eggs (n = 30): Length: 97.6 ± 3.3 (90–102) μm; maximum width 40.9 ± 1.5 (37–43) μm; Length/Width = 2.4 ± 0.09 (2.2–2.5). Egg shell hyaline and unsculptured when observed under light microscope. Second-stage juvenile in full embryonated eggs folded three to four times.

#### Male

Body vermiform, tapering anteriorly; tail rounded, with twisted posterior body portion. Lip region slightly set off from body, labial cap relatively small, labial disc not elevated. Lip framework strong and sclerotized, vestibule extension distinct. Prominent slit-like amphidial openings between labial disc and lateral lips. In SEM view, the labial disc is slightly narrower and raised above the merged subventral and subdorsal medial lip sectors, with a centred oval prestoma into which opens a slit-like dorso-ventrally oriented stoma; lateral lips margins rounded. Lip region moderately high and lacking annulation. Stylet delicate and straight, with cone and shaft broadening slightly in the distal part. Stylet knobs mostly rounded, laterally or obliquely directed, merging gradually with the base of the shaft. Lateral field consisting of four incisures with areolations along body but only few actually cross central field. Procorpus distinctly outlined, 4.1–5.2 times the length of metacorpus. Metacorpus ovoid, with a strong valve apparatus. Excretory duct curved. Excretory pore distinct and usually located three to four annuli posterior to hemizonid. Testis single, long, monorchic, occupying 40 to 57% of body length. Tail usually curved ventrally, short, with bluntly rounded tip and finely annulated. Spicules of variable length, arcuate and with two pores clearly visible at tip. Gubernaculum distinct. Phasmids small and located at level of cloacal aperture.

#### Second-stage juveniles

Body vermiform, tapering slightly towards posterior end. Lip region narrower than body and slightly set off. Labial cap slightly elevated. Lip framework weakly developed. Labial disc and medial lips fused. In labial disc, a stoma-like slit located in an ovoid prestoma and surrounded by six inner labial sensilla. In SEM view, the labial disc appears oval to rectangular in shape, raised above medial lips, to which it merges in a dumbbell-shaped structure. Lip region smooth and lacking annulation. Amphidial apertures elongated and located between labial disc and lateral lips. Body annulated from anterior end to terminus. Lateral field consisting of four incisures, with areolations along body. Stylet delicate, with cone straight, narrow, sharply pointed, shaft almost cylindrical, and knobs small, rounded, laterally directed. Pharynx with a long, cylindrical procorpus (4.0–5.0 times length of metacorpus), round-oval metacorpus, short isthmus and rather long gland lobe, with three equally sized nuclei and overlapping intestine ventrally. Hemizonid located anterior to excretory pore, extending for ca. two body annuli. Excretory pore located posterior to nerve ring. Excretory duct curved and discernible when it reaches intestine. Rectum slightly dilated. Tail short, conoid, tail terminus broad and rounded at the tip, with several constrictions in the hyaline region. Tail annulation fine, regular in the proximal two-thirds, becoming slightly coarser and irregular in the distal part. Hyaline tail terminus clearly defined and short, phasmids small, difficult to observe.

#### Measurements, morphology and distribution

Morphometric variability is described in Tables 5 and 6 and morphological traits shown in Figs 2–7. In addition to the type locality, *Meloidogyne oleae* sp. nov. was collected from the rhizosphere and root systems of rosebush, lesser periwinkle, and carob tree, all of them located in the type locality, and rhizosphere and roots of cultivated olive in Antequera, Málaga province, being restricted to this region in Andalusia (Table 1, Fig 1). The nematode population densities in these host-plants were 152.87 eggs + J2s/g root of rosebush, 0.75 eggs + J2s/g root of lesser periwinkle, and 63.40 eggs + J2s/g root of carob tree, confirming that all of them can be considered as alternative host.

#### Relationships

The female perineal pattern morphology of *M*. *oleae* sp. nov. is mostly rounded-oval with dorsal arch generally low, which places it in Jepson’s perineal pattern-Group 3 [64], and second-stage juveniles with tail short belonging to morphospecies tail shape-Group 2 [64], is morphometrically close by tail length to some members of tail shape-Group 1 as *M*. *brevicauda* Loos, 1953 [65], *M*. *nataliei* Golden, Rose & Bird, 1981 [66], and *M*. *indica* Whitehead, 1968 [67] from which it differs clearly by perineal pattern morphology. From *M*. *brevicauda*, it differs by female stylet (12.5–14.0 μm *vs* 22 μm in *M*. *brevicauda*), second-stage juvenile tail (23.0–28.5 μm *vs* 17.5–28.0 μm in *M*. *brevicauda*), male stylet (13.5–18.0 μm *vs* 19.5–21.0 μm in *M*. *brevicauda*), and spicules (21.0–32.0 μm *vs* 34.0–42.5 μm in *M*. *brevicauda*) [67]. From *M*. *nataliei* differs by second stage juvenile stylet (11.0–13.0 μm vs 21.9–22.8 μm in *M*. *nataliei*), female (445–790 μm vs 731–1247 μm in *M*. *nataliei*), female stylet (12.5–14.0 μm vs 21.1–22.4 μm in *M*. *nataliei*), more anterior position of excretory pore (11.5–13.5 μm vs 6–11 μm in *M*. *nataliei*) and shorter spicule (21.0–32.0 μm vs 41.3–44.3 μm in *M*. *nataliei*). From *M*. *indica* differs by second-stage juvenile stylet and tail lengths (11.0–13.0 μm, 23.0–28.5 μm *vs* 13.0–20.0 μm, 10.0–14.0 μm in *M*. *indica*) [67]. It can be separated from other *Meloidogyne* spp. parasitizing olive, such as *M*. *lusitanica* [68] by clear differences in perineal pattern, having a medium to high trapezoidal dorsal arch and distinct punctuations in the tail terminus region, female stylet length (12.5–14.0 μm *vs* 16.0–19.0 μm), excretory pore/stylet length ratio (0.9–1.0 *vs* 1.6–3.8), second-stage juvenile and male stylet length (11.0–13.5 μm, 13.5–18.0 μm *vs* 13.0–16.0 μm, 21.0–27.0 μm, respectively), second-stage juvenile tail length (23.0–28.5 μm *vs* 39.0–50.0 μm), and isoenzyme phenotype [68].

In addition, *M*. *oleae* sp. nov. is molecularly related to *M*. *artiellia* Franklin, 1961 and *M*. *baetica*, but can be clearly differentiated from them in having a distinct perineal pattern (mostly rounded-oval with dorsal arch generally low *vs* very distinctive pattern formed by striae and ridges pronounced near vulva), female stylet length (12.5–14.0 μm *vs* 12–16 μm, 17.0–19.0 μm, respectively), excretory pore/stylet length ratio (0.9–1.0 *vs* 1.5–2.3, 0.5–0.8, respectively), second-stage juvenile tail length (23.0–28.5 μm *vs* 23.0–26.0 μm, 47–54 μm, respectively) [15, 69, 70].

### Isozyme analysis

The isozyme electrophoretic analysis of five-specimens of young egg-laying females of *M*. *oleae* sp. nov. revealed one very slow weak A1 Est band after repeated and prolonged staining (Fig 8) and a N1c Mdh phenotype with a very weak-staining band (Fig 8) that did not occur in the Est and Mdh phenotypes of *M*. *javanica*, which showed J3 and N1 phenotypes, respectively (Fig 8) or in other Est and Mdh phenotypes previously identified for other *Meloidogyne* spp.

### Molecular divergence of the new species

Blast search of the three ribosomal markers used in this study for *M*. *oleae* sp. nov. (MH011963-MH011983) revealed the highest similarity values with the accessions correspond to *M*. *artiellia* and *M*. *baetica* being 84 and 81% similar for the D2-D3 (KY433424, AY150369 and AY150367), 79% similar for the ITS (KC545880, AF248477 and AY150366) and finally 92% similar for the partial 18S rRNA (KC875391, KC875392 and MH011982), respectively. For mtDNA, five *cox*II-16s and two *cox*I were sequenced (MG996751-MG996755, MG996758-MG996759), which showed similarity values of 78% with *M*. *aberrans* Tao, Xu, Yuan, Wang, Lin, Zhuo & Liao, 2017 and from 74% to 76% with several accession deposited in GenBank, including the new accession of *M*. *baetica* (MG996756) obtained in this study. Unfortunately, no data is available for the *cox*II-16S region from *M*. *artiellia*. Blast search of the *cox*I sequences from *M*. *oleae* sp. nov. (MG996758-MG996759) revealed similarity values ranging from 84 to 89% with all *cox*I accessions from *Meloidogyne* spp. deposited in GenBank. Intraspecific variation among the eight D2-D3 sequences from *M*. *oleae* sp. nov. included in this study was 9 nucleotides and 1 indel, 11 nucleotides and 1 indel among for the four ITS sequences, 12 nucleotides and no indels for the five *cox*II-16S studied, and finally, no variation found for the three partial 18S and *cox*I region.

### Histopathology of *Meloidogyne oleae* sp. nov. in wild and cultivated olives and other host-plants

*Meloidogyne oleae* sp. nov. established permanent, fully developed feeding sites on wild and cultivated olives (Fig 9), as well as other host-plant such as rosebush, and lesser periwinkle (Fig 10), and females reached to maturity and produced egg-masses containing 1 to 248 eggs. Both wild and cultivated olives and the other host-plants showed a similar disease reaction. Galls induced by *M*. *oleae* sp. nov. on olive roots were variable in size but relatively small (almost two times the root diameter), and were commonly located along the root axis but also on the root tip. Usually galls contained more than one nematode female. Occasionally an egg mass was found inside the root cortical tissue, but the majority of egg masses were observed at the root surface. Comparative histological observation of healthy and *M*. *oleae* sp. nov. infected roots showed cellular alterations in the cortex, endodermis, pericycle, and vascular parenchyma induced by the nematode. Permanent feeding site development caused the typical vascular damage due to the presence of the nematode and the multinucleate giant cells (Figs 9 and 10). That is, in the permanent feeding sites, the nematode induced formation of large multinucleate giant cells adjacent to the vascular tissues. Nematode feeding sites comprised three to eight giant cells that surrounded the lip region of a single female. This formation led to disruption and reduction of xylem elements and primary phloem cells. Active multinucleated giant cells contained granular cytoplasm, thickened cell wall, and hypertrophied nuclei and nucleoli. The histological modifications induced by *M*. *oleae* sp. nov. in roots also revealed a typical susceptible reaction to infection by the nematode.

### Phylogenetic relationships of *Meloidogyne oleae* sp. nov. within the genus *Meloidogyne*

**Fig 11:**
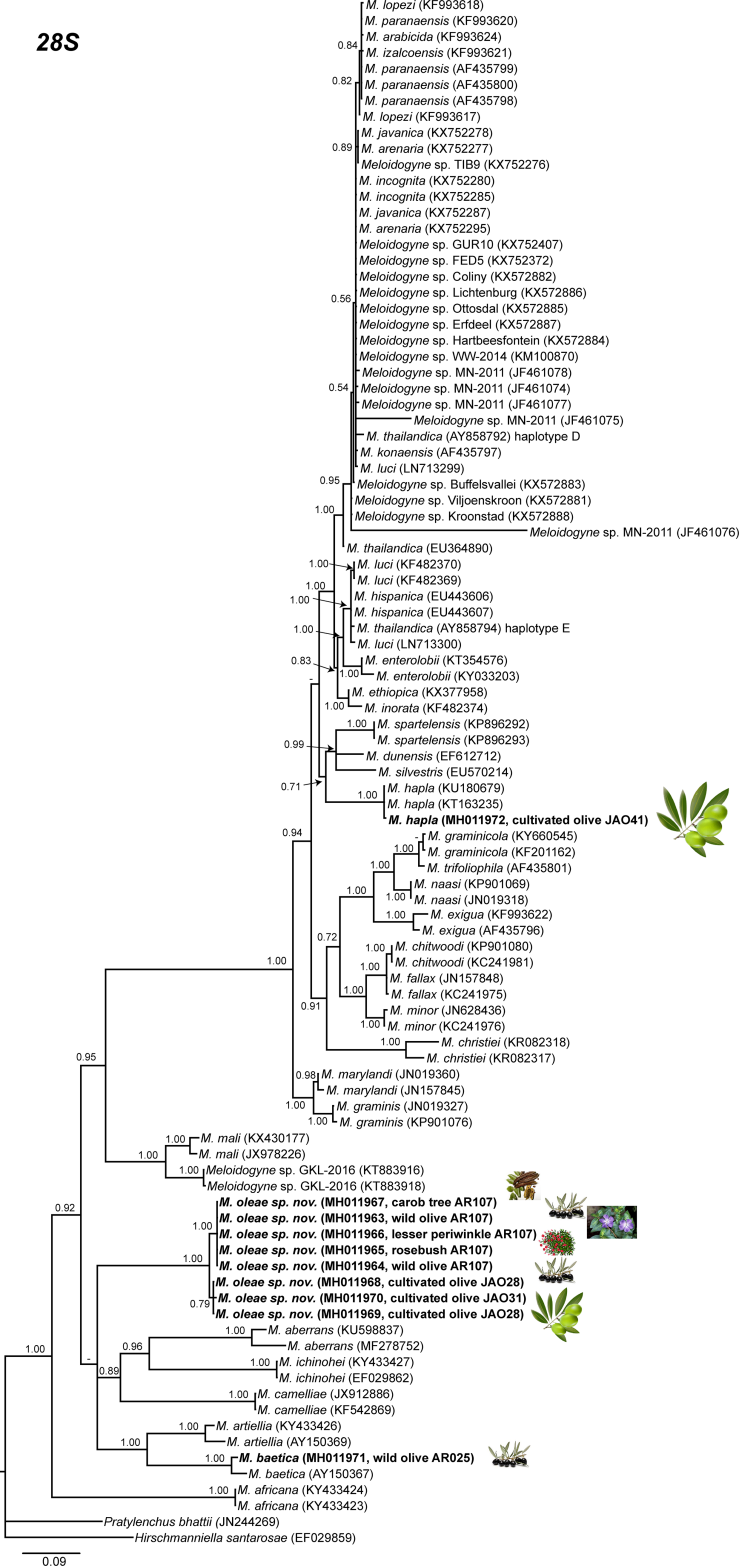
The 50% majority rule consensus tree from Bayesian inference analysis generated from the D2-D3 of 28S rRNA gene dataset of *Meloidogyne* spp. with the TIM3+I+G model. Posterior probabilities more than 0.70 are given for appropriate clades. Newly obtained sequences are in bold letters. Scale bar = expected changes per site.

**Fig 12:**
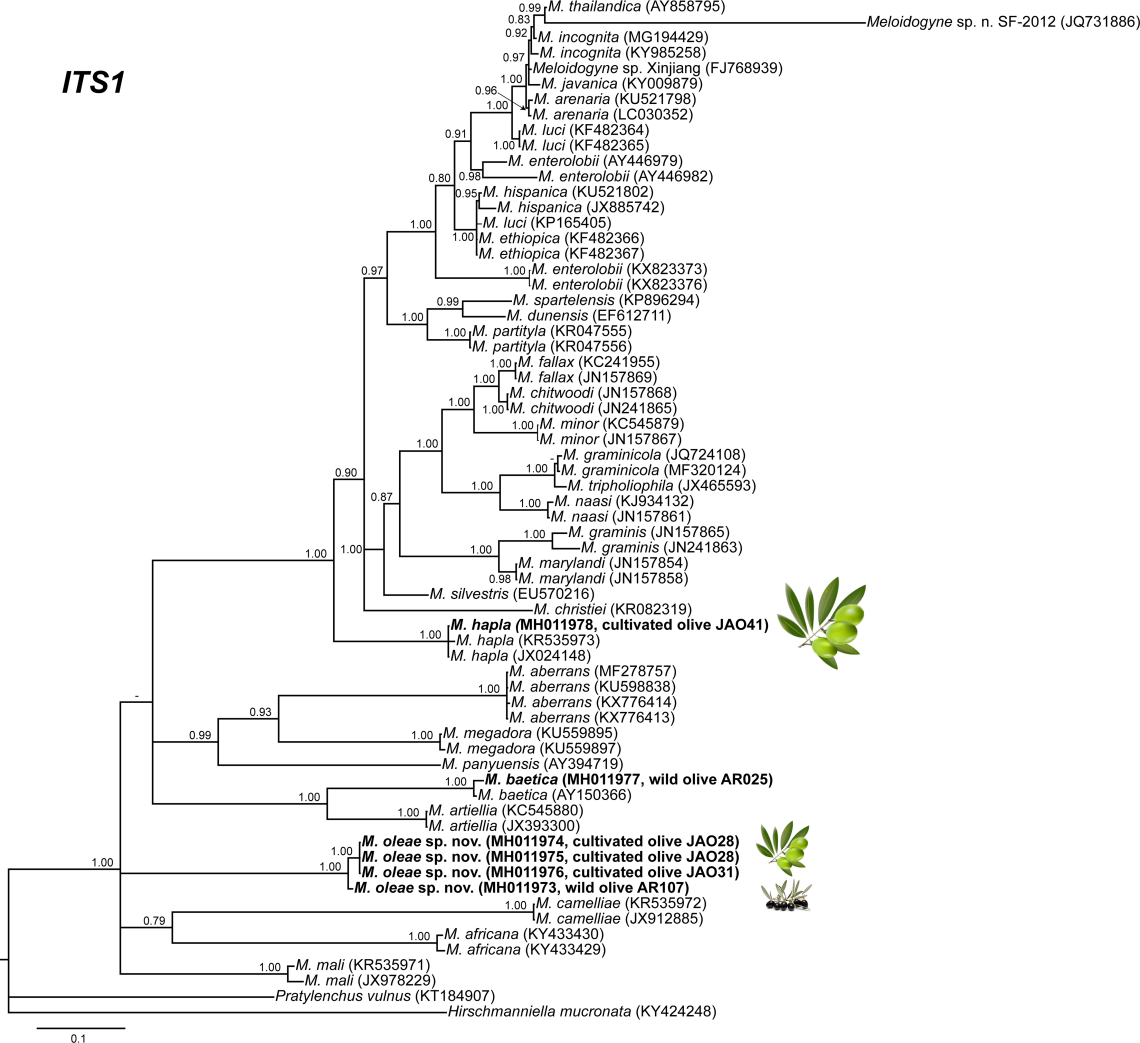
The 50% majority rule consensus trees from Bayesian inference analysis generated from the ITS rRNA gene dataset of *Meloidogyne* spp. with the TIM2+I+G model. Posterior probabilities more than 0.70 are given for appropriate clades. Newly obtained sequences are in bold letters. Scale bar = expected changes per site.

**Fig 13:**
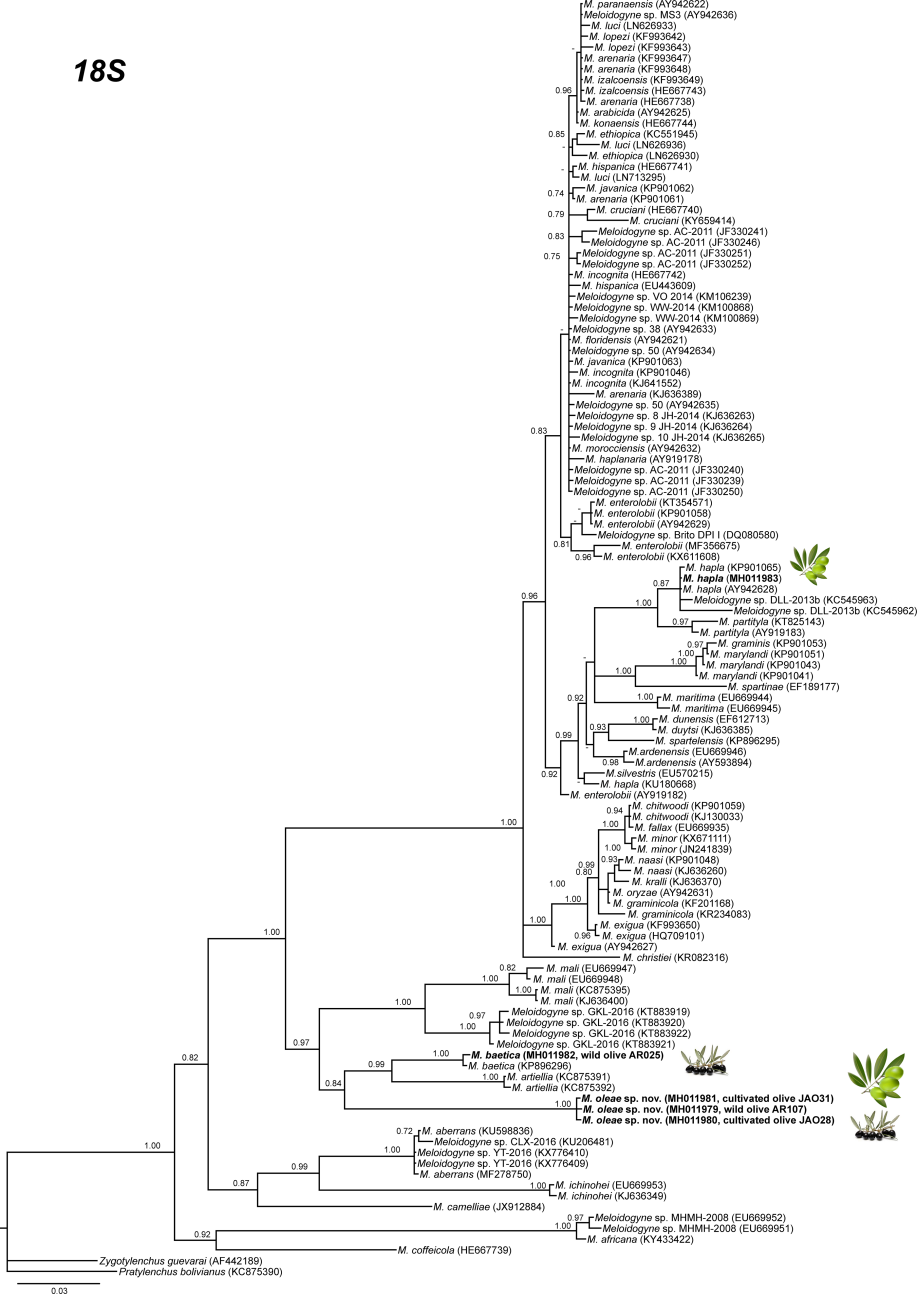
The 50% majority rule consensus trees from Bayesian inference analysis generated from the partial 18S rRNA gene dataset of *Meloidogyne* spp. with the GTR+I+G model. Posterior probabilities more than 0.70 are given for appropriate clades. Newly obtained sequences are in bold letters. Scale bar = expected changes per site.

**Fig 14:**
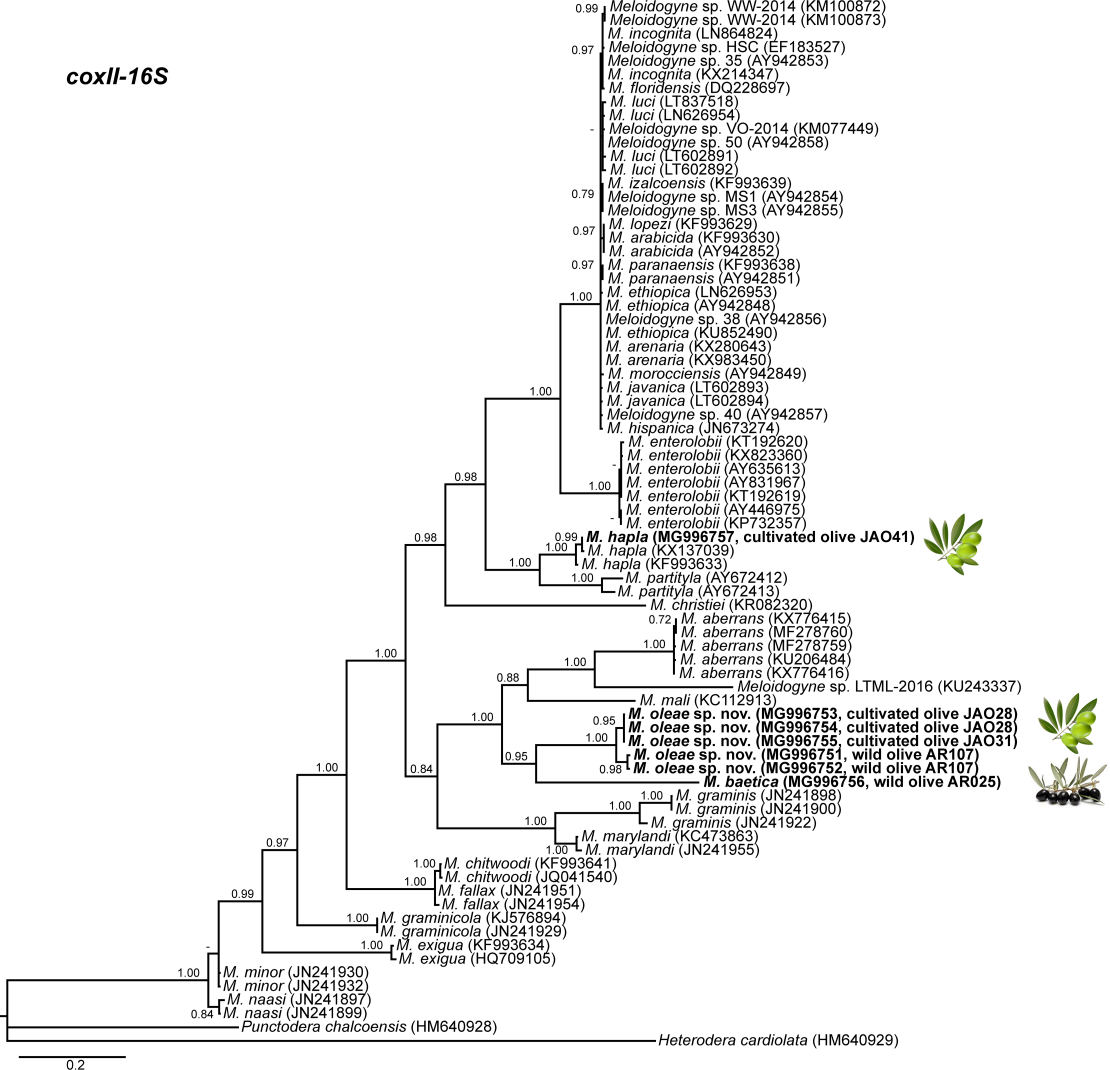
The 50% majority rule consensus trees from Bayesian inference analysis generated from the partial *cox*II-16S mtDNA gene dataset of *Meloidogyne* spp. with the TVM+I+G model. Posterior probabilities more than 0.70 are given for appropriate clades. Newly obtained sequences are in bold letters. Scale bar = expected changes per site.

**Fig 15:**
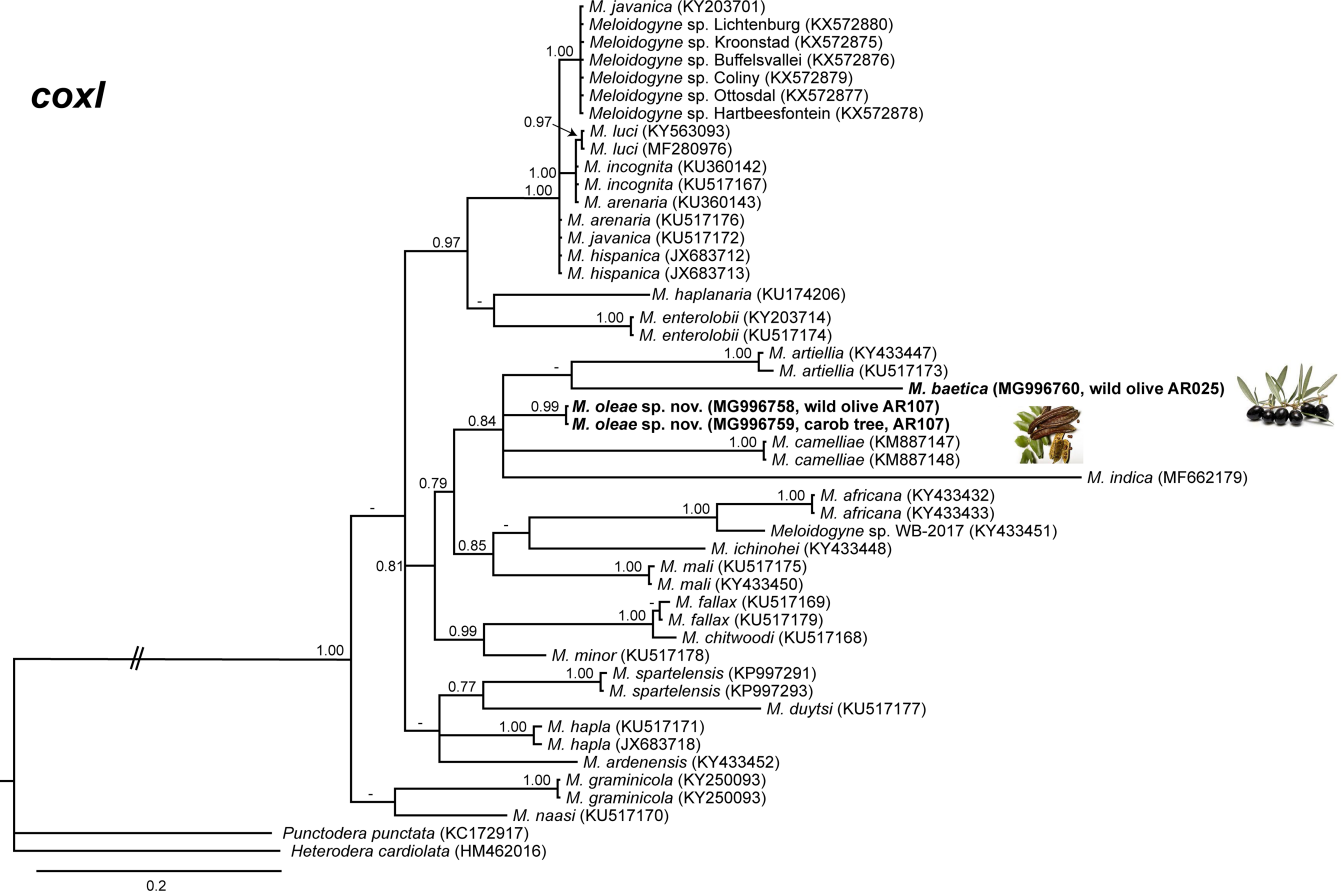
The 50% majority rule consensus trees from Bayesian inference analysis generated from the partial *cox*I gene dataset of *Meloidogyne* spp. with the GTR+G model. Posterior probabilities more than 0.70 are given for appropriate clades. Newly obtained sequences are in bold letters. Scale bar = expected changes per site.

The amplification of D2-D3 expansion segments of 28S rRNA, ITS1 rRNA, 18S rRNA, *cox*I and partial *cox*II-16S genes yielded a single fragment of approximately 700, 750, 1600, 400 and 500 bp, respectively, based on estimation using gel electrophoresis. Sequences from other species of *Meloidogyne* spp. obtained from National Center for Biotechnology Information (http://www.ncbi.nlm.nih.gov/) were used for further phylogenetic studies. The 50% majority rule consensus 28S rRNA gene BI tree of *Meloidogyne* spp. (Fig 11) based in a multiple edited alignment including 97 sequences and 691 total characters showed several clades, the superior well-supported clade (Posterior Probability (PP) = 1.00) clustered the majority of *Meloidogyne* spp. with available D2-D3 sequences, except for *M*. *oleae* sp. nov. (MH011963-MH011970), *M*. *aberrans* (KU598837, MF278752), *M*. *africana* Whitehead, 1960 (KY433423, KY433424), *M*. *artiellia* (AY150369, KY433426), *M*. *baetica* (AY150367, MH011971), *M*. *camelliae* Golden, 1979 (JX912886, KF542869), *M*. *ichinohei* Araki, 1992 (EF029862, KY433427) and *M*. *mali* Ito, Oshima & Ichinohe, 1969 (KX430177, JX978226) which occupied basal clades. However, the phylogenetic relationships among these species were not well resolved. The BI trees inferred from analyses of the partial 18S rRNA and ITS region (including 116 and 66 sequences, respectively) showed a similar topology than D2-D3 tree (Figs 12 and 13), a superior clade which include the most of accessions from *Meloidogyne* spp. and a basal part where appeared some of the species mentioned above, such as *M*. *aberrans*, *M*. *artiellia*, *M*. *baetica*, *M*. *camelliae* and *M*. *ichinohei* and *M*. *oleae* sp. nov. For the partial 18S, *M*. *oleae* sp. nov. (MH011979-MH011983) clustered with *M*. *baetica* (MH011982, KP866296) and *M*. *artiellia* (KC875391-KC875392) in a moderately supported clade (PP = 0.84), instead of ITS region where *M*. *oleae* sp. nov. (MH011973-MH011978) clustered alone in a well-supported clade (PP = 1.00). However, the position of some *Meloidogyne* spp. that occupied the basal position in the tree was not well-resolved for this region. Finally, the 50% majority rule consensus tree generated with the mtDNA genes *cox*I and *cox*II-16S alignment by BI analyses is presented in Figs 14 and 15. For this genes, some of species, such as *M*. *graminis* (Sledge & Golden, 1964) Whitehead, 1968 (JN241900, JN241898, JN241922), *M*. *graminicola* Golden & Birchfield, 1965 (JN241929, KJ576894), *M*. *naasi* Franklin 1965 (JN241899, JN241897), *M*. *minor* Karssen et al. 2004 (JN241930, JN241932), *M*. *exigua* Göeldi, 1892 (HQ709105, KF993634), *M*. *fallax* Karssen 1996 (JN241951, JN241954) and *M*. *chitwoodi* Golden, O’Bannon, Santo & Finley 1980 (KF993641, JQ041540) occupied different position in the trees in comparison to the trees formed with rRNA markers. However, *M*. *oleae* sp. nov. (MG996751-MG996755) keep a similar position as in the trees formed with D2-D3 and partial 18S rRNA, clustering with *M*. *baetica* (MG996756) in a well-supported clade (PP = 0.95) for the *cox*II-16S and with *M*. *artiellia* (KU517173-KY433447) and *M*. *baetica* (MG996760) for the partial *cox*I in a moderately-supported clade (PP = 0.84).

### Explanatory variables driving community patterns of RKNs on cultivated olives

In spite of the broad range of explanatory variables included in all four data set, community composition of RKNs was only significantly shaped by 3 predictors, explaining one third of the variation in community composition of RKNs (Table 7). Agriculture management was the most influent data set followed by soil contributing by 21.49% and 6.55% to explain variation in community composition, respectively. Interestingly, variables considered within both climate and topography data set were not associated with community patterns of RKNs (Table 7).

**Table 7:** Forward selection procedure results of ecological predictors in explaining community patterns of root-knot nematodes (RKNs) infesting soils of olive orchards from cultivated olive in Andalusia (southern Spain).

Explanatory variables^1^^,^ ^2^^,^^3^^,^^4^	*R*^*2*^	*R*^*2*^_cum_	*R*^*2*^_adj cum_	*P value*	*Variation explained (%)* ^5^
**Agronomic management**					
*Alley*					
Cover vegetation	0.1516	0.1516	0.1215	0.0116	12.15
*Irrigation*					
Irrigated	0.1346	0.2862	0.2149	0.0444	9.34
**Soil**					
*Soil texture*					
Sandy loam)	0.1080	0.3943	0.2804	0.0429	6.55
**All**	**-**	**-**	**0.2804**	**-**	**28.04**

^1^ We used as ecological predictors the explanatory variables included in the climatic, soil, agronomic management and topography data sets as whole.

^2^ Order of explanatory variables is based on the *R*^*2*^ values.

^3^ See Table 2 for details of explanatory variables.

^4^ Forward selection procedure was performed with the indications described in Materials and Methods section).

^5^ Percentage of variation explained by explanatory variables are given as *R*^*2*^ adjusted (*R*^*2*^_adj_ *100).

Cover vegetation on alley class was the most influent variable accounting for 12.15% of the variation (Table 7). This finding indicates that this factor could play a key role on community patterns of RKNs infesting soils from cultivated olive. Irrigated class was another influential agriculture management practices, which contributed by 9.34% of variation of community composition. Uniquely soil texture was the soil factor accounted for that variation (Table 7), with sandy loam type being the only one with 6.55% of variation explained.

**Fig 16:**
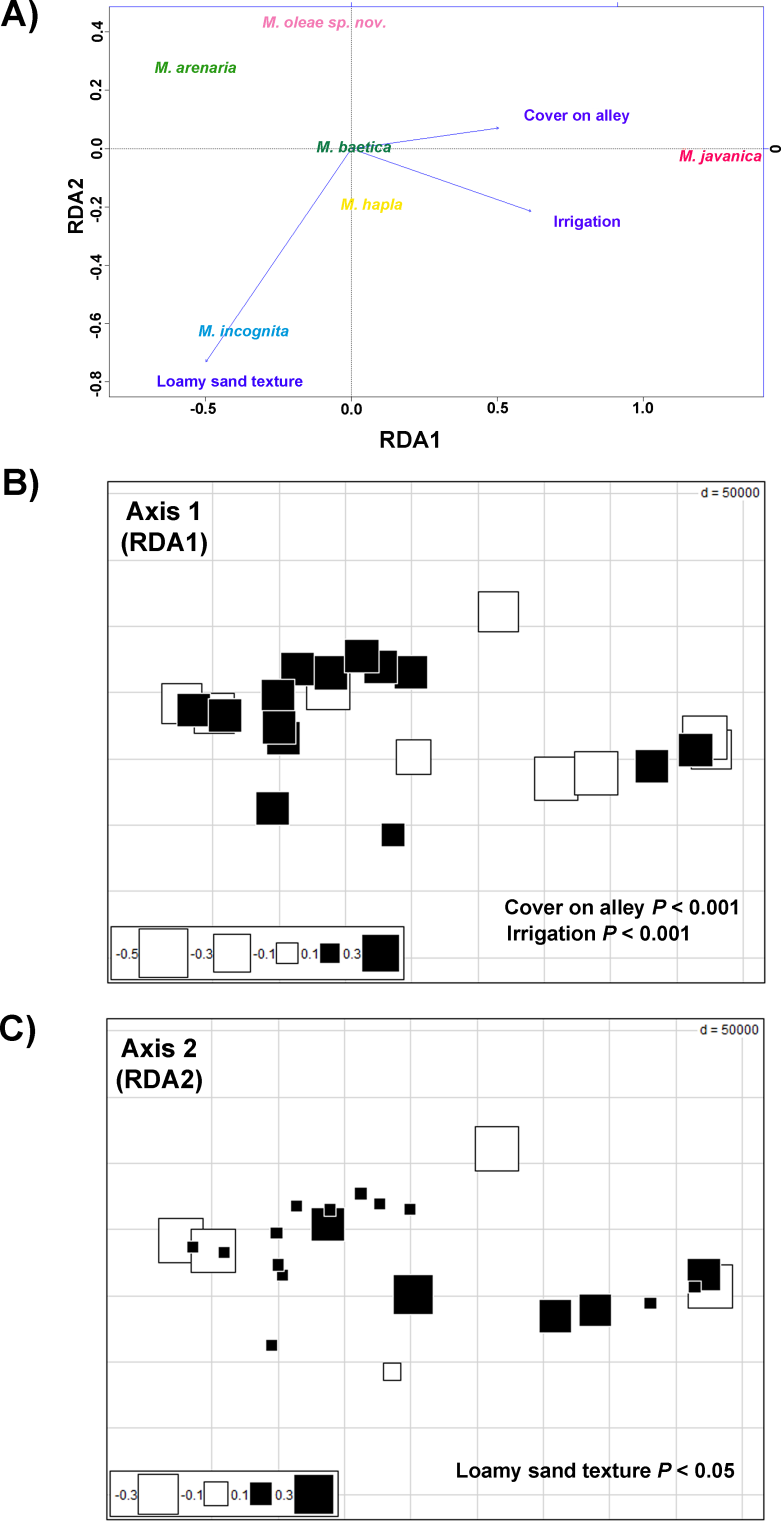
Canonical ordination analyses of Hellinger-transformed prevalence *Meloidogyne* spp. data on cultivated olives with respect to environmental variables. The response variables are the fitted explanatory variables from the four data set (i.e. climate, soil, topography and agronomic management) by forward selection procedure. A) Ordination plots of the redundancy analyses used to investigate the relationships between prevalence of *Meloidogyne* spp. and explanatory variables. Ecological predictors are represented on the plots as agronomic practices related with irrigation regimen (Irrigation) and cover vegetation on alley (Cover on alley), and soil loamy sand texture (Loamy sand texture) on olive orchards. The first canonical axis (RDA axis 1) explains 85.9% of species-variables relationships, and the second axis (RDA axis 2) explains 11.2%. B) Trend-surface analysis of the canonical axis 1 of the RDA analysis. Spatial structure variation related with agronomic practices including Irrigation (*P*<0.001) and Cover on alley (*P*<0.001) variables. C) Trend-surface analysis of the canonical axis 2 of the RDA analysis. Spatial structure variation related with soil properties including soil loamy sand texture (*P*<0.05). For trend-surface analyses, fitted sampling site scores from RDA are plotted on geographical coordinates. Sampling sites with the same colours show similarly trends of relationships between Hellinger-transformed prevalence of *Meloidogyne* spp. data and explanatory variables of cultivated olives; symbol size indicates the level of similarly (i.e. small symbols for low similarity, and large symbols for high similarity. Interpretation of the spatial variation with respect to explanatory variables was performed using regression analyses of the two canonical axes on the environmental variables after normality tests.

Redundancy analysis (Fig 16A) found that variation of community composition of RKNs among olive orchards was associated with the three fitted explanatory variables (Table 7). Three canonical axes were retained for accounting 33% of the total of variation in species composition, and 97.5% of the explained variation (that accounted for the three explanatory variables). We also determined relationships between species and explanatory variables. In fact, it should be noted that *M*. *incognita* was closely related with loamy sand texture class (Fig 16A). It indicates that the presence of this RKN species could be strongly associated with soil composed by coarse particles. On the other hand, the distribution of *M*. *javanica* was associated with agronomic management practices such as cover vegetation on alley and irrigated regimen on olive orchards (Fig 16A). On the contrary, ordination diagrams of RDA showed that *M*. *arenaria* was located in an opposite trend to irrigation variable (Fig 16A). No close relationships were found between the rest of *Meloidogyne* spp. detected and explanatory variables.

Finally, trend-surface analysis showed that RKNs community on cultivated olives was significantly spatially structured (Fig 16B and 16C). Linear models performed on the relationships between canonical axes of RDA and explanatory variables revealed significant associations among them. Canonical axis 1 was significantly related with both irrigation regimen (*P* < 0.001) and cover vegetation on alley (*P* < 0.001) variables, and axis 2 was associated by soils with loamy sand texture (*P* < 0.05). Overall, these findings suggested that the spatial variation of community of RKNs on cultivated olives is strongly explained with the three fitted explanatory variables (Fig 16B and 16C). In addition, spatial scale of the influence of agronomic practices (i.e. irrigation and management on alley) was larger than that found for soil texture. It agrees with the higher proportion of variation explained by management variables (Table 7).

## Discussion

This study aimed to obtain knowledge and a better understanding of the occurrence, abundance and biodiversity of root-knot nematodes of the genus *Meloidogyne* associated with wild and cultivated olives in Andalusia, southern Spain, including the environmental variables associated with their distribution and their molecular phylogeny. An extensive and systematic nematological survey was conducted that included 123 and 376 sampling sites from wild and cultivated olives, respectively. We found 35 populations of *Meloidogyne* spp. infesting olive soils. We describe a new *Meloidogyne* species, enlarging the diversity of *Meloidogyne* species in the Iberian Peninsula infecting olive and other hosts. This new species potentially constitutes an additional threat for olive in this area, but further studies are necessary in order to confirm this assumption as the observed levels of infection are not very high. This new species evidences the hidden biodiversity in the olive crop, as it has been demonstrated with the recent data obtained for the genus *Meloidogyne* in Morocco and the description of *M*. *spartelensis* in the olive rhizosphere [16]. Our results are the first reporting the prevalence of *Meloidogyne* in the most important olive growing area worldwide, with more than 1.5 x 10^6^ ha [71] and show the importance of *Meloidogyne* spp. in this area with presence in 6.6% of the sampled points. These findings differ from a recent report by Ali *et al*. [16], in which the incidence for wild, feral and cultivated olives in Morocco was 12.2% and *M*. *incognita*, *M*. *baetica* and *M*. *oleae* sp. nov. were absent. On the contrary the other species they indicate, *M*. *javanica*, *M*. *hapla* and *M*. *arenaria*, were also found in our study. Additionally, their analyses reported important diversity in electrophoretic esterase patterns not found in our study (data not shown).

In our case, three sampled points (Table 1) reached damaging threshold levels in olive trees, ranging from 0.49 to 0.9 eggs or juveniles/cm^3^ of soil for *M*. *javanica* [72]. Other studies have shown a significant reduction of main shoot-length growth by 37.6% and 10.7% at 0.1 and 12.8 juveniles/cm^3^ soil of *M*. *javanica* and *M*. *incognita*, respectively, in comparison to uninfected plants [73]. These studies depend highly on the source of inoculum as juveniles alone are usually more damaging than juveniles and eggs, as well as the susceptibility of the studied cultivar. The olive trees studied in our sampling did not show any symptomatology associated with the presence of root-knot nematodes, but the damage caused by these nematodes would likely have been more significant in fields re-planted with younger trees in soil with high levels of inoculum. Additionally, as the majority of our sampling sites were in plantations established many years ago, biocontrol agents could reduce the damage levels of RKNs by a suppressive effect on the disease after long term association with the presence of the disease in the soil [74]. In fact, some *Pasteuria* sp. infections were detected in male paratypes (Fig 6). Nor were the highest RKN densities in soil correlated with the highest densities in the olive roots. This could be explained by the differences in suitability and reproduction in alternative hosts such as weeds for the different RKNs species [75], or to variability in reproduction levels among RKN species and olive cultivars, as has been demonstrated in other studies [76].

One of the likely major sources of root-knot nematode inoculum in the field could be the use of infected rooted plantlets, as suggested by other authors [19, 28]. In Spain, a study in olive nurseries reported higher percentages of root-knot nematodes in infected plantlets [*Meloidogyne incognita* (14.7%), *M*. *javanica* (11.2%), and *M*. *arenaria* (2.7%)] [28] compared to our results in cultivated fields. Similar results were obtained between RKN species found in olive nurseries and their distribution in olive fields in Morocco [19]. Hamza *et al*. [19] suggested that the presence of *M*. *incognita* in nurseries alone but not in orchards might be due to either the competition from other plant-parasitic nematodes or unfit local habitats. In our case, the presence of *M*. *incognita* in four provinces showed the possible adaptability of this species to the olive habitat, which moreover belonged to cultivated olive in all cases where the nematode was found. Interestingly, the province of Jaen, one of the most important Andalusian olive growing areas and characterized by a majority of years-old traditional olive orchards, had only two samples infected with RKNs. This could indicate the human involvement in spreading the disease in recent times to new areas when new plantations are established using rooted plantlets with associated soil as is usual in the new oliviculture.

To explain the observed species distribution we performed a robust statistical analysis, including a large number of variables and considering only the statistically important ones for discussion. The studied data showed only a few statistically significant variables for the distribution of *Meloidogyne* spp. in cultivated olive in Southern Spain: alley [alley (cover vegetation), irrigation, and soil texture (particularly sandy loam). The presence of *M*. *incognita* was highly correlated with sandy loamy soils, the presence of *M*. *javanica* with agronomical factors (irrigated soils and cover vegetation present) while the presence of *M*. *arenaria* was correlated with the absence of alley cover vegetation and unirrigated fields (Fig 16). These data could explain that our species distribution in olive orchards is mainly based on ecological factors. The competition among species as suggested by Hamza *et al*. [19] would be more difficult to determine. In fact, only two sampling points had a mixture of species (*M*. *javanica* and *M*. *hapla*), probably due to reasons indicated above such as few introductions and biocontrol agents available in the field, suggested by Hamza *et al*. [19] and others. Surprisingly these sampling points were in warm environments close to the sea (Table 1). *Meloidogyne hapla* is usually associated with temperate regions [77] or high altitude [78], and in the study of Ali *et al*. [16] was detected in the coldest regions in northern Morocco also characterized by high annual rainfall. Our data suggest that while the loamy sandy soil texture only influenced some specific sampling points (Fig 16C), the influence of agronomic factors was important for all positive sampled points (Fig 16B). In particular, both the frequent use of vegetative cover in Andalusia to protect against erosion, improve soil structure and increase organic matter contents [79] and of irrigation to increase olive yield, could encourage the establishment and high population levels of *M*. *javanica*.

*Meloidogyne oleae* sp. nov. increased the knowledge of biodiversity of *Meloidogyne* species associated with olive or with its rhizosphere (*M*. *arenaria*, *M*. *baetica*, *M*. *javanica*, *M*. *incognita*, *M*. *hapla*, *M*. *lusitanica*, *M*. *spartelensis*) [9, 15, 16]. In this sense the presence of a new species associated with the glacier refugia (“Serrania de Ronda” plant refuge area) inside the Mediterranean regional hotspot 2 [80] would be similar to the recently discovered species associated with the rhizosphere of olive in Morocco (specifically in the “Rif Mountains” plant refuge area) as *M*. *spartelensis* [16] and *M*. *baetica* in Southern Spain (specifically in the “Cadiz/Algeciras region” plant refuge area) [15]. The restricted location of *M*. *oleae* sp. nov. to three points in Southern Spain could be due to their origin in this area or by restricted ecological and competition conditions, in this sense, olive seems one of other putative hosts for this species, as it also infects rosebush, lesser periwinkle and carob tree, in all cases as a weak pathogen. This RKN species showed molecular markers, feeding site anatomy, and parasitic features similar to *M*. *artiellia*, but distinct to the range of non-olive hosts such as cereals and legumes to which *M*. *artiellia* is adapted [81]. Significantly, giant cells from *M*. *oleae* sp. nov. showed giant nuclei similar to the ones produced by *M*. *artiellia* (few and bigger than other species of *Meloidogyne*) [82]. *Meloidogyne baetica*, a specific parasite of olive [15] is geographically and phylogenetically related to *M*. *oleae* sp. nov., also parasitic on olive, but the morphological differences between these two species are considerable. The specificity of both nematodes in this area in Southern Spain, however, suggests their possible evolution from a common ancestor.

The majority of the identified *Meloidogyne* spp. in the olive rhizosphere was previously characterized molecularly, but this study provides additional new molecular markers for partial 18S, *cox*I and *coxII-*16S of *M*. *baetica*, the others region studied, D2-D3 and ITS, were obtained from the same individual and matched well with other sequences from *M*. *baetica* deposited in the GenBank. The amplification of the mtDNA fragment followed by digestion with HinfI consistently discriminated populations of *M*. *arenaria* from *M*. *incognita* and *M*. *javanica* [31]. The majority of the *Meloidogyne* species showed congruence in the phylogenetic relationships within D2-D3, ITS1, and partial 18S, although this congruence was not maintained in the case of mtDNA including partial *cox*II and 16S probably, due to the rapid evolution of this molecule in comparison to relative nuclear rRNA genes [83]. The molecular data of *M*. *oleae* sp. nov. obtained in this work clearly support the identity of *M*. *oleae* as a new species.

## Conclusions

In summary, this study provides new insights into the diversity and prevalence of the genus *Meloidogyne* associated with wild and cultivated olive tree in southern Spain, with the description of a new species (*M*. *oleae* sp. nov.) parasitizing both forms of olive in addition to other hosts. Three major parameters drive the distribution of *Meloidogyne* in cultivated olive in southern Spain, which are alley vegetative cover, irrigation and loamy sandy soil texture. Very likely these parameters induce the selection of *Meloidogyne* species from major dispersal sources, such as nursery -rooted plantlets. However competition among species cannot be completely excluded, although we found only two of the 33 infected sampling points were co-infested by two *Meloidogyne* species (*M*. *javanica* and *M*. *hapla*). The observed populations ranged from low to high levels in few fields, but no damage symptoms associated with the disease were observed in the infested orchards. Major damage, however, could likely occur in fields with some of the detected levels, such as in cases of young plants or with the new intensive agriculture frequent in Spain which uses irrigation, heavy mechanization, and high density plantations, all of which have been shown highly conducive for *M*. *javanica*.
